# What helps the helpers? Resilience and risk factors for general and profession-specific mental health problems in psychotherapists during the COVID-19 pandemic

**DOI:** 10.3389/fpsyg.2023.1272199

**Published:** 2023-12-18

**Authors:** Matthias Zerban, Lara Marie Christine Puhlmann, Dana Lassri, Peter Fonagy, P. Read Montague, Natalia Kiselnikova, Nicolas Lorenzini, Alex Desatnik, Raffael Kalisch, Tobias Nolte

**Affiliations:** ^1^Neuroimaging Center (NIC), Focus Program Translational Neuroscience (FTN), Johannes Gutenberg University Medical Center, Mainz, Germany; ^2^Leibniz Institute for Resilience Research (LIR), Mainz, Germany; ^3^Max Planck Institute for Human Cognitive and Brain Sciences, Leipzig, Germany; ^4^Research Department of Clinical, Educational and Health Psychology, University College London, London, United Kingdom; ^5^The Paul Baerwald School of Social Work and Social Welfare, The Hebrew University of Jerusalem, Jerusalem, Israel; ^6^Anna Freud National Centre for Children and Families, London, United Kingdom; ^7^Virginia Tech Carilion Fralin Biomedical Research Institute, Virginia Polytechnic Institute and State University, Roanoke, VA, United States; ^8^Department of Psychosocial Studies, Birkbeck College, University of London, London, United Kingdom; ^9^Open Door Young People’s Service, London, United Kingdom

**Keywords:** stress, adversity, mentalizing, positive reappraisal, self-compassion, mental health professionals, mental health practitioners, compassion satisfaction

## Abstract

**Introduction:**

Although the COVID-19 pandemic has severely affected wellbeing of at-risk groups, most research on resilience employed convenience samples. We investigated psychosocial resilience and risk factors (RFs) for the wellbeing of psychotherapists and other mental health practitioners, an under-researched population that provides essential support for other at-risk groups and was uniquely burdened by the pandemic.

**Method:**

We examined 18 psychosocial factors for their association with resilience, of which four were chosen due to their likely relevance specifically for therapists, in a cross-sectional multi-national sample (*N* = 569) surveyed between June and September 2020. Resilience was operationalized dimensionally and outcome-based as lower stressor reactivity (SR), meaning fewer mental health problems than predicted given a participant’s levels of stressor exposure. General SR (SR_G_) scores expressed reactivity in terms of general internalizing problems, while profession-specific SR (SR_S_) scores expressed reactivity in terms of burnout and secondary trauma, typical problems of mental health practitioners.

**Results:**

Factors previously identified as RFs in other populations, including perceived social support, optimism and self-compassion, were almost all significant in the study population (SR_G_: 18/18 RFs, absolute βs = 0.16–0.40; SR_S_: 15/18 RFs, absolute βs = 0.19–0.39 all *P*s < 0.001). Compassion satisfaction emerged as uniquely relevant for mental health practitioners in regularized regression.

**Discussion:**

Our work identifies psychosocial RFs for mental health practitioners’ wellbeing during crisis. Most identified factors are general, in that they are associated with resilience to a wider range of mental health problems, and global, in that they have also been observed in other populations and stressor constellations.

## Introduction

The COVID-19 pandemic has triggered unprecedented interest in its mental health consequences in both the general population and at-risk groups, including health care workers. Meta analyses and systematic reviews identified significant, but moderate rises in mental health disorders and distress in the general population, in particular depression and anxiety symptoms (e.g., Manchia et al., 2022; Riepenhausen et al., 2022; Robinson et al., 2022). More severe mental health consequences have been observed in specific subgroups. For example, meta-analyses suggest that one quarter to one third of health care workers reported clinically meaningful symptoms of depression or anxiety during the pandemic (Pappa et al., 2020; Al Maqbali et al., 2021). This pattern may be exacerbated by additional individual risk factors or lack of resources (Crocamo et al., 2021; Lassri et al., 2022; Manchia et al., 2022). On the flipside, although health care workers generally appear to be at heightened risk due to their specific work challenges, these detrimental effects may also be attenuated by their personality characteristics, coping behaviors, and other resources (De Brier et al., 2020).

### Mental health practitioners in the pandemic

Among health care workers, mental health practitioners are one under-researched group experiencing unique stress load during the pandemic (Aafjes-van Doorn et al., 2020, 2021a). Examining risk and resilience factors in psychotherapists and other mental health professionals is especially important given that these factors might not only impact therapists’ experience of professional self-doubt, secondary traumatic stress, and general mental health, but also impact the quality of care they are able to provide to their patients. Consequently, therapists’ mental health is of importance to mental health in the broader population.

It is well documented that therapists’ individual characteristics play an important role in psychotherapy processes and treatment outcomes (Lutz et al., 2007; Wampold and Owen, 2021). Therapist factors or traits include the therapist’s coping patterns, personality and interpersonal patterns, attachment characteristics and mentalizing capacity, or emotion regulation (Cologon et al., 2017; Lingiardi et al., 2018; Heinonen and Nissen-Lie, 2020). Less is known, however, about how these factors relate to therapists’ own wellbeing and resilient responding in times of heightened stressor exposure.

The practice of psychotherapy is known to be stressful for therapists (Briggs and Munley, 2008; Luther et al., 2017). Therapists’ way of coping has been shown to impact effectiveness and, in maladaptive cases, to also increase vulnerability to these stress reactions (Simionato and Simpson, 2018). Some therapists even experience patient-contingent compassion fatigue or burnout (e.g., O'Connor et al., 2018; Simionato and Simpson, 2018).

Beyond this general burden, the COVID-19 pandemic has challenged psychotherapists in an unprecedented way (e.g., Aafjes-van Doorn et al., 2020, 2021a). This ongoing public health challenge has negatively impacted mental health in both general and clinical populations (Clemente-Suárez et al., 2021; Robinson et al., 2022), and a rise in burnout prevalence has been observed in psychotherapists (Van Hoy et al., 2022; Mittal et al., 2023) with more complex trajectories longitudinally (Van Hoy and Rzeszutek, 2023). Particularly when therapists and patients are simultaneously experiencing a disaster, e.g., during Hurricane Katrina (Culver et al., 2011) or following the terrorist attacks on 9/11 (Boscarino et al., 2004), the experience of secondary traumatic stress seems to increase the disaster’s deleterious impact on mental health practitioners (Aafjes-van Doorn et al., 2020). When exposed to higher levels of patient distress related to the pandemic, combined with sudden professional transitions that were required, such as switching to online therapy, in addition to their own personal and family coping, therapists might fall back on using lower-level, less mature psychological defense mechanisms or coping strategies (Aafjes-van Doorn et al., 2021b). Aafjes-van Doorn et al. (2022) recently found that during the pandemic therapists’ professional self-doubt was both higher than pre-pandemic and predicted by higher secondary traumatic stress and weaker working alliance with patients, less clinical experience, and less acceptance of online therapy technology.

Overall, while the effects of the pandemic on general populations and mental health practitioners more broadly is well documented, few studies have investigated the impact on psychotherapists (e.g., Aafjes-van Doorn et al., 2020, 2021a,b, 2022; Probst et al., 2020). Findings converge on higher levels of distress or symptomatology. Yet little is known about the nature of protective factors that may play a role in mitigating COVID-19-related distress for therapists.

### Operationalizing resilience

Resilience has been defined as the maintenance or rapid recovery of mental health and psychosocial functioning during and after times of adversity (Bonanno et al., 2015; Kalisch et al., 2017) and can be operationalized as an outcome to adversity (Kalisch et al., 2021). To approximate outcome-based resilience in a cross-sectional design during the initial COVID-19 pandemic, Veer et al. (2021) have earlier proposed to relate participants’ deterioration in mental health, retrospectively reported over a time window of 2 weeks, to their stressor exposure reported for that same time window. This provides a norm relationship between stressors and mental health problems in the study cohort, to which an individual’s mental health problems can be related using a residualization approach. Participants with more mental health problems than would be expected given their own level of stressor exposure thus show high “stressor reactivity” (SR) and can be classified as less resilient during the reporting time window, while participants with comparatively fewer problems than expected show relatively lower SR and can be classified as more resilient. This method controls for individual differences in stressor exposure, which may otherwise confound estimates of resilience, and thus increases comparability of the mental health outcome between different individuals and populations (Kalisch et al., 2021; Veer et al., 2021). In the current study, we first calculated a general stressor reactivity score (SR_G_) for general internalizing mental health problems, using the norm relationship between internalizing problems and stressors, in line with our previous work. Second, to account for the specific burden experienced by psychotherapists (i.e., profession-specific mental health problems), we developed a novel profession-specific SR score (SR_S_) that indicates norm deviations in professional quality of life with aspects of burnout and secondary traumatic stress, thus inversely approximating profession-specific resilience.

### Resilience and risk factors

Resilience factors have been defined as social, psychological, and biological factors positively associated with outcome-based resilience (Kalisch et al., 2017). Kalisch et al. (2015) have introduced the category of ‘general’ resilience factors, which are factors that protect not just against single, but against a wider range of mental health problems and therefore are especially interesting targets for preventative interventions. Calculating two different resilience outcomes that diverge according to the type of mental health problems they integrate allowed us to test whether a given factor would negatively associate with SR both in terms of general (SR_G_) and profession-specific mental health problems (SR_S_). Such a factor could then be considered general. Another category of resilience factors introduced by Kalisch et al. (2015) is the category of ‘global’, or universal, resilience factors. These protect not only against various stressor-induced mental health impairments (i.e., are general) but they are also helpful against the detrimental effects of different stressors or problem constellations or can be observed to be active in various stressed populations. Global resilience factors would be even more promising intervention targets.

Previous work has identified resilience factors in other specific circumstances and populations (e.g., Ehret et al., 2015; Zessin et al., 2015; Veer et al., 2021). Extending this work, we here assessed how these potential psychosocial resilience factors were associated with outcome-based resilience in a specific group of *N* = 569 practicing mental health practitioners, largely comprising psychotherapists and clinical psychologists, affected by the COVID-19 pandemic. Apart from assessing resilience factors relevant to this specific population, this also allowed us to ask whether previously identified resilience factors could also be classified as global. We considered it conceivable that previously identified resilience factors may also be important for our study population, because they may be universally helpful in situations of high stressor exposure. Conversely, the professional, personal, and training background of mental health practitioners may also lead them to rely on other factors that are specifically available to, or particularly relevant for, this population (e.g., Klasen et al., 2019; Rogoff et al., 2021). Empirical evidence for the latter so far remains scarce and mostly relates to therapist factors associated with patient outcome. We therefore also tested a set of factors taken form earlier research on therapists (Stamm, 2010; Wilkerson and Basco, 2014; Lingiardi et al., 2018) for (a) whether they also classify as resilience factors for therapists’ mental wellbeing in this sample, and (b) if so, whether they are general (i.e., negatively associated with both SR_G_ and SR_S_).

Finally, next to including factors that we expected to be positively associated with resilience (protective or resilience factors, such as optimism) we also tested factors we expected to be negatively associated with resilience (risk factors, such as neuroticism; Veer et al., 2021), to gain a broader overview of therapists’ resources and vulnerabilities. These resilience and risk factors are collectively referred to as ‘RFs’ in the remainder. We examined associations of 18 psychosocial RFs with therapists’ general and profession-specific SR scores.

### Hypotheses

The selected RFs firstly comprised a set of factors investigated for their association with resilience against internalizing problems (inverse SR_G_) by Veer et al. (2021) in a convenience sample in the wider population during the first wave of the COVID-19 pandemic. Here, we tested these factors’ protective influence for this specific population and profession-specific symptoms, and by extension, their general and global nature. They included perceived positive appraisal style (PAS) as well as related constructs capturing positive stressor appraisal tendencies, like optimism (OPT), which should all be positively associated with resilience (negatively associated with SR) according to Positive Appraisal Style Theory of Resilience (PASTOR; Kalisch et al., 2015). Specifically, we employed the process-focused perceived positive appraisal style (PAS_p_; Petri-Romão et al., 2023) scale that assesses the tendency to use cognitive operations (emotion regulation processes) that generate positive appraisal contents in stressful situations. Other RFs from this set were perceived social support (PSS), good perceived stress response recovery (REC), neuroticism (NEU) as risk factor, and positive appraisal specifically of the Corona crisis (PAC).

Secondly, we investigated a set of RFs less studied in the context of the pandemic (e.g., Schäfer et al., 2022). We added self-compassion (SCO) as a potential general and global RF to our assessment. According to Neff (2003), a person’s level of self-compassion relies on the interplay between the distinct components of self-kindness (SKI) versus self-judgment (SJU), a sense of common humanity (COH) versus isolation (ISO), and mindfulness (MIN) versus over-identification (OVI). SCO has been linked to wellbeing and lower levels of psychopathology (Ehret et al., 2015; Zessin et al., 2015) and the effectiveness of compassion-based interventions has been demonstrated (Ferrari et al., 2019). Self-criticism (SCR) was also added to the assessment as a potential risk factor, as it is present in various psychopathologies (Werner et al., 2019) and often discussed as being related to SCO in depression research (e.g., Wakelin et al., 2022). None of these factors have been tested for their association with resilience to different types of mental health problems in the same stressor-exposed population nor for their effectiveness in pandemic-exposed mental health practitioners.

As a last set, we examined the role of RFs that may be particularly important for this professional group (profession-relevant RFs), namely self-efficacy as a therapist (TSE), compassion satisfaction (COS), and mentalizing (operationalized as certainty [CER] and uncertainty [UNC] about mental states), that is, the capacity to make sense of experiences, including stressors, in terms of intentional mental states. The latter has been shown to mitigate developmental adversity (e.g., Huang et al., 2020) and to be linked to resilience in the general population (Maerz et al., 2022; Tohme et al., 2022). Studies have shown that psychotherapists tend to have higher levels of mentalizing capacity (Klasen et al., 2019; Rogoff et al., 2021) and that therapist’s mentalizing abilities play a role in treatment outcomes in psychotherapy (Cologon et al., 2017).

We also asked whether PAS_p_ would statistically mediate the hypothesized association between PSS and SR, aiming to replicate Veer et al. (2021). This tested the claim by Positive Appraisal Style Theory of Resilience (PASTOR; Kalisch et al., 2015) that even RFs that are not restricted to the cognitive domain unfold their influence on resilience depending on how they shape the appraisal of stressful situations. While social support – the embeddedness of an individual in its social network – is an external RF, it will only influence resilient responding if it is appraised as helpful and reliable. Similarly, we tested whether REC mediates the association between PAS_p_ and SR. These analyses tested, as proposed in PASTOR, that habitual positive appraisal eventually promotes resilience by facilitating optimal stress response regulation (Kalisch et al., 2015).

## Participants and methods

### Participants

Data for the present study were collected between June 2020 and November 2020 across the United Kingdom, Israel, Russia, and North and Latin America in English, Hebrew, and Russian via Qualtrics,^1^ a secured web-based survey data collection system. Clicking on the link to the anonymous survey guided potential respondents to a page providing information about the study and a consent form. Inclusion criteria were participants’ profession (currently practicing psychotherapy, being a psychotherapy trainee, or working therapeutically in related psychological professions including social work or counseling), practice of clinical work during the pandemic, and a minimum age of 18 years. Participants were recruited via social media (Facebook and WhatsApp), professional mailing lists and clinician interest groups, using a snowball technique. Preliminary power calculation for initially planned analyses (which included the possibility of separate analyses based on language subgroups) resulted in a required sample size of 1,000 participants, such that data collection was stopped after 1,089 respondents had used the survey link. Required sample size specifically for the present analyses was then calculated *a posteriori*. The expected effect sizes for this power analysis were conservatively based on the estimated association between SR and behavioral coping style (BCS), the predictor with the smallest standardized beta coefficient (*β* = 0.11) in a previous population-based study of which most RFs were derived (Veer et al., 2021). This power analysis arrived at a minimum required sample size of 299 participants for a power of 80% power and 352 participants for 90%, respectively. Participants gave their informed consent online and electronically during data collection. They were not financially reimbursed for their participation. The study was approved by the Ethical Review Board of The Paul Baerwald School of Social Work and Social Welfare, The Hebrew University of Jerusalem and was conducted in accordance with the Declaration of Helsinki.

### Stressors and mental health problems (components of SR scores)

#### Stressor exposure

The questionnaire used to assess stressor exposure provides a detailed assessment of stressors participants had been exposed to in the past 2 weeks (see Supplementary Table S1). This includes exposure to general stressors (11 broad classes of stressors such as negative political events, family conflicts, or mental health problems), and to stressors specific to the Corona crisis (29 items such as having COVID-19 symptoms, loss of social contact, or problems arranging childcare). The list was developed for studies on psychological resilience during the COVID-19 pandemic by the project “DynaMORE – Dynamic Modelling of Resilience” (Veer et al., 2021; Bögemann et al., 2022). Stressor exposure was quantified as the sum count of the reported general and COVID-19-specific stressors, weighted by their rated severity, as reported previously (Veer et al., 2021).

#### Mental health problems

Two conceptually distinct types of mental health symptoms were assessed: First, as in Veer et al. (2021), we measured general mental health problems (P_G_) using the 12 items version of the general health questionnaire (GHQ-12), a widely used screening tool for internalizing mental health issues in the general population (Goldberg et al., 1997). It captures symptoms of anxiety, depression, insomnia, social problems as well as somatic symptoms and was employed as an indicator of general inability to carry out normal functions. This measure allows us to compare mental health problems in the present specific sample to the wider population.

Second, we measured participants’ mental health problems related to their profession as a mental health practitioner (P_S_) by means of the professional quality of life scale (ProQoL; Stamm, 2010) and the secondary traumatic stress scale (STSS; Bride et al., 2004; Bride, 2013). The ProQoL scale assesses three domains of helpers’ work experiences termed compassion satisfaction (i.e., the fulfillment and pleasure therapists may derive from effectively helping their patients; COS), burnout (i.e., a response to chronic emotional and interpersonal work-related stressors characterized by loss of interest in work, exhaustion of personal resources, and decline of enthusiasm; CF_BO), and secondary traumatic stress (i.e., posttraumatic symptoms experienced by therapists when witnessing the trauma/stress of those they support; CF_STS). Burnout and secondary traumatic stress jointly reflect compassion fatigue. Notably, the compassion satisfaction subscale (COS) is not a measure of symptoms or problems, and we instead hypothesized that the experience of work-related compassion satisfaction would qualify as a potential RF. The STSS is designed to specifically measure secondary traumatic stress and was included as an additional measure for this type of mental health problems. While there is some overlap between the CF_STS and the STSS, both instruments were included as the latter more specifically captures symptomatology in line with the DSM-5 (Stamm, 2010; Bride, 2013).

To determine whether these four (sub-)scales would best be grouped into one common measure of helper-specific mental health problems (single component), or rather describe different aspects of mental health problems (multiple components), we performed a principal component analysis (PCA) on the sum scores of the aforementioned scales. Parallel analysis was conducted to determine the number of components. The resulting component(s) would then be used for calculation of SR_S_ (see below). The PCA analysis also allowed us to assess the relevance of compassion satisfaction compared to other ProQoL subscales for P_S_. High loading on one or several components with other symptom scales would suggest that compassion satisfaction was strongly tied to compassion fatigue/secondary traumatic stress and should be included as an inverse indicator of P_S_; weaker loadings would suggest that compassion satisfaction does not simply measures inverse symptoms, as hypothesized, and may be evaluated as a potential resilience factor (predictor).

### Calculation of SR scores (dependent variables)

As an approximation to resilience, SR scores were computed via normative modeling (for a conceptual description of this methodology, see Kalisch et al., 2021). Participants’ mental health problem scores (P_G_, P_S_) were first regressed onto stressor exposure scores (E), establishing the sample’s normative E-P relationship (average SR). P and E scores were z-standardized before analysis. Subsequently, the deviation of each participant’s (average) P score from this norm relationship (i.e., its regression residual) was calculated, which expresses participants’ individual mental health reactivity to their current stressor exposure. A positive SR score reflects higher-than-predicted reactivity, whereas a negative SR score reflects lower-than-predicted reactivity. A low SR score can be considered a dimensional approximate measure of outcome-based resilience.

In the present work, two different types of SR scores were calculated, based on the two different measures of mental health symptoms. SR_G_ was derived from residuals of the P_G_ ~ E regression and reflects the relative degree to which participants react to stressor exposure with internalizing symptoms common in the general population. SR_S_ was derived from residuals of the P_S_ ~ E regression and is introduced here for the first time. It reflects the relative degree to which mental health practitioners display profession-specific mental health problems in relation to their current exposure to general stressors.

### Resilience and risk factors and covariates (independent variables)

Detailed descriptions of all measured variables and instruments are provided in Supplementary Table S1.

#### Covariate candidates

Demographic and physical health variables included age and gender, as well as geographic, educational, and social variables. Health status variables included current or previous mental health diagnosis, as well as COVID-19 risk and infection status.

#### Resilience and risk factors

We examined associations of SR_G_ and SR_S_ with 14 RFs derived from Veer et al. (2021), Neff (2003) and Rudich et al. (2008), and four RFs that we assumed to be particularly relevant to the profession of a therapist (Stamm, 2010; Wilkerson and Basco, 2014; Fonagy et al., 2016) but potentially also general, that is, associated with both SR_G_ and SR_S_. Administration of all instruments was preceded by clear instructions for filling out the respective measure. Likert scale items were verbally anchored to reduce response bias.

### Data cleaning and preparation

Of the 610 participants who completed the questionnaire, 38 were excluded due to missing values in the questionnaires required to compute the outcome measures (i.e., stressor list, GHQ-12, STSS, ProQoL). For statistical reasons, we also excluded participants who reported demographic characteristics with exceptionally low frequencies compared to the rest of the sample, which would have led to an unreliable selection of covariates (see also Veer et al., 2021; Bögemann et al., 2022). This was the case for the gender category “diverse,” which was indicated by three participants after data cleaning.

Our final sample consisted of *N* = 569 participants (*n* = 488 [86%] female, mean age = 43.58 years [SD = 11.85]). Among these, 201 provided fully complete datasets including all RFs and covariates. A large proportion of missing data points specifically for Corona-related social support, general self-efficacy and behavioral coping style (BCS) measures stemmed from a technical failure due to which these scales were not included in the Hebrew version of the questionnaire. With 197 missing data points, BCS showed the largest proportion of missingness These three scales thus eventually had to be excluded from analysis. Additionally, some questionnaires allowed for optional responses (detailed information about missingness can be found in the Supplementary Figures S1, S2). To deal with the resulting significant proportion of missingness with however wide distribution across the sample, multiple imputation was chosen (Van Buuren, 2018; see Supplementary material section S1 for more information). Analyses were then based on all imputations by either pooling results for all imputed datasets or, in case of the regularized regression analysis, stacking the data. Multiple regression results for non-imputed data are shown in the Supplementary Figure S4. All independent variables were centered and scaled before running the statistical models. To test model assumptions, visual checks of residual distributions were performed (see Supplementary Figure S5).

### Statistical analyses

#### Covariate selection

To determine relevant socio-demographic covariates for parsimonious modeling, we first assessed the influence of the socio-demographic and health variables on the SR scores using separate univariate regression analyses. In line with Veer et al. (2021), covariate candidates surviving a likelihood-ratio test at *p* < 0.2 were included in the analyses. Age and gender were included in all models.

#### Hypothesis-driven analyses

Subsequently, our directed hypotheses about RFs were tested using separate multiple regressions with covariates to assess the effects of each RF on the two different SR scores, as in Veer et al. (2021). Mediation analyses were conducted following a Baron and Kenny approach (Baron and Kenny, 1986) and the distribution-of-the-product method was used to determine indirect paths (see Veer et al., 2021; Bögemann et al., 2022). To account for multiple comparisons within each analysis family (multiple regressions and mediation analyses with each of SR_G_ and SR_S_), a Bonferroni-corrected alpha level of *p* < 0.05/18 = 0.0028, two-tailed, was used for multiple regression analyses and *p* < 0.05/2 = 0.025 for mediation analyses (Bonferroni, 1936). Effect sizes were indicated through fully standardized beta estimates.

#### Exploratory combined multi-variable analysis (regularized regression)

Most of the assessed RFs are conceptually related and generally show considerable simple and partial correlations (Supplementary Figure S3 for zero correlations). To identify which of the assessed RFs may be particularly good predictors of SR, we examined their relative strengths of association with SR_S_ and SR_G_ compared to all other RFs. Of the self-compassion scales only the compound scale SCO and not the subscales were included for reasons of high collinearity. RFs and the included covariates were jointly entered in an elastic net regression analysis, which combines elements of lasso and ridge regression (Hastie et al., 2015). Both hyperparameters α and λ were determined by cross-validation. Detailed information can be found in the Supplementary section S2.

### Software

Data cleaning and statistical analyses were performed in R (v4.1.0).^2^ Multiple imputation was done with the R package mice (v3.8.0; Van Buuren and Groothuis-Oudshoorn, 2011) and elastic net analyses were conducted with the glmnet package (v4.1–1; Friedman et al., 2010).

## Results

### Sample characteristics

Following data cleaning (see below), *N* = 569 usable and complete data sets were available from the initial 1,089 responses (For characteristics of the final sample, see Supplementary Table S2). Most of the sample defined themselves as women (86%), were married, in a domestic partnership or civil union (58%) and not living alone (87%), worked in the health care system (72%) with a broad range of average household income, mostly considered themselves healthy (94%) and were neither belonging to a COVID-19 risk group (79%) nor suffering from a mental health diagnosis (81%). This final sample comprised clinical psychologists (*n* = 200), psychotherapists (*n* = 194), educational and school psychologists (*n* = 37), social workers (*n* = 26), psychoanalysts (*n* = 24), art and music therapists (*n* = 12), medical/rehabilitation/counseling psychologists (*n* = 12), psychiatrists (*n* = 11), and other mental health practitioners (*n* = 53).

### Profession-specific mental health problems

In a first analysis step in developing a profession-specific outcome measure, we determined the components of helper-specific mental health problems (P_S_) for the calculation of SR_S_. A principal component analysis (PCA) with the ProQoL subscales compassion satisfaction (COS), compassion fatigue: burnout (CF_BO), compassion fatigue: secondary trauma stress (CF_STS), and the STSS indicated a single component with equally strong positive loadings for CF_BO, CF_STS and STSS, and weaker negative loadings for COS (see Supplementary Table S3). Further, COS loaded strongly on the second component, while the other variables did not. Accordingly, COS was not a strong inverse indicator of profession-specific mental health problems, and we treated it as a potential RF instead. We refit the PCA without COS (see Table 1), again resulting in a single component, which was subsequently used to calculate SR_S_, in line with our residualization approach.

**Table 1:** Results of principal component analysis for profession-specific stressor reactivity.

	Components		
	1	2	3
Importance of Components			
Eigenvalues	2.24	0.51	0.24
Proportion of variance	0.75	0.17	0.08
Cumulative proportion	0.75	0.92	1.00
Component loadings			
STSS	0.61	0.26	0.75
ProQoL: CF_STS	0.59	0.49	−0.64
ProQoL: CF_BO	0.53	−0.83	−0.15

STSS, Secondary Trauma Stress Scale; CF_STS, Compassion Fatigue: Secondary Trauma Stress; CF_BO, Compassion Fatigue: Burnout; ProQoL marks scales of the Professional Quality of Life Questionnaire.

### E-P relationship and SR scores

Linear regression analyses were conducted to derive the SR scores, regressing the mental health problems (P_G_ or P_S_) on the stressor exposure score E. Both models were significant overall [P_G_: *F*(1, 567) = 114, *p* < 0.001, *R^2^* = 0.17; P_S_: *F*(1, 566) = 86.02, *p* < 0.001, *R^2^* = 0.13] and both P variables showed a significant relationship with E (P_G_: *β* = 0.41, *p* < 0.001; P_S_: *β* = 0.36, *p* < 0.001). The resulting SR scores, SR_G_ and SR_S_, only correlated moderately at *r* = 0.52, suggesting they each captured a unique part of the variance. While including a second order polynomial term did not improve the model fit for the E-P_G_ relationship significantly, a significantly better fit for the quadratic model was found with P_S_. However, since no difference in results was observed, for the sake of comparability, reported SR scores were based on the linear regression.

### Covariate selection

Of the covariate candidates, age, gender, current relationship status, people living in household, clinical experience in years, perceived health condition, and diagnosis of mental disorder passed the likelihood-ratio test at *p* < 0.2 and thus were included in the further analyses (see Supplementary Table S4).

### Multiple regression analyses

**Figure 1:**
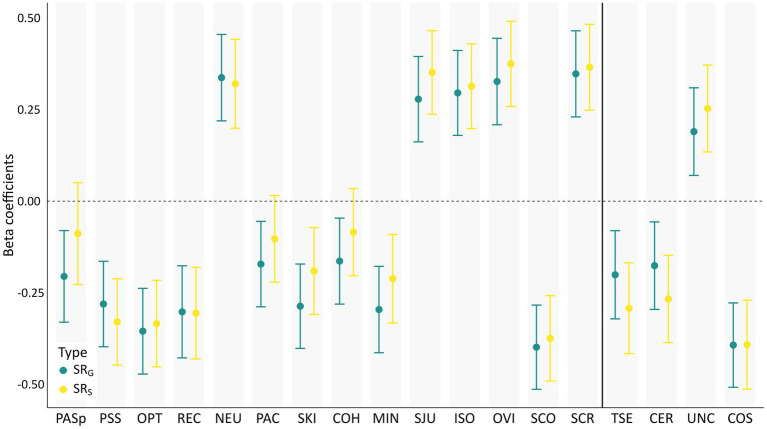
Associations between hypothesized resilience and risk factors and stressor reactivity. Shown are standardized beta coefficients of resilience factors (RFs) explaining stressor reactivity (SR) in multiple regressions, calculated separately for each RF. Coefficients of RFs from the set of Veer et al. (2021) are displayed on the left, profession-relevant RFs are placed on the right. Covariates age, gender, current relationship status, people in household, and clinical experience in years are included in each model. PASp, perceived positive appraisal style – process-focused; PSS, perceived social support; OPT, optimism; REC, perceived good stress recovery; NEU, neuroticism; PAC, perceived positive appraisal of the Corona crisis; SKI, self-kindness; COH, common humanity, MIN, mindfulness; SJU, self-judgment; ISO, isolation; OVI, overidentification; SCO, self-compassion; SCR, self-criticism; TSE, self-efficacy as a therapist; CER, certainty about mental states; UNC, uncertainty about mental states; COS, compassion satisfaction. Error bars depict 99% Confidence intervals.

Beta coefficients for associations between each RF and both SR_G_ and SR_s_ in the separate regression analyses are depicted in Figure 1. Most RFs show strikingly similar associations with both outcome measures, indicated by near-identical beta coefficients and largely overlapping confidence intervals. This indicates that the tested RFs can be classified as general (Kalisch et al., 2015), that is, they are relevant across various mental health domains in stressor-exposed individuals. However, perceived positive appraisal style – process-focused (PAS_p_), perceived positive appraisal specifically of the Corona crisis (PAC), and common sense of humanity (COH) exhibited slightly weaker associations with SR_S_ than with SR_G_, leading to significant results only for the latter. At an uncorrected alpha level of *p* < 0.05 PAC and COH were significantly associated with SR_S_. Thus, reduction of statistical power due to Bonferroni correction potentially led to insignificant results for these RFs. However, very small effect sizes (Cohen’s *f*^2^ = 0.01 for PAS, PAC and COH) indicated low relevance of these RFs in explaining variance in SR_S_ (Cohen, 1988). Detailed results of the multiple regression analyses can be found in the Supplement (SR_G_: Supplementary Tables S5–S7; SR_S_: Supplementary Tables S8–S10). Multiple regression analyses without multiple imputation showed comparable results for all RFs (see Supplementary Figure S4).

To investigate the effect of SR_S_ above and beyond SR_G_, we ran all multiple regression models for SR_S_ with SR_G_ as control variable. Results are depicted in Supplementary Figure S7. As to be expected, beta coefficients decreased when SR_G_ was included. The overall picture, however, remained unchanged and only SKI and MIN were no longer significantly associated with SR_S_ with SR_G_ included.

### Mediation analyses

The predicted negative mediation of the association of perceived social support (PSS) and SR by PAS_p_ was found for SR_G_ (est. mediation path: −0.03, 97.5% CI [−0.08, −0.001]) but not SR_S_, in line with above non-significant effect of PAS_p_ in the SR_S_ regression analysis.

By contrast, the expected negative mediation of the association between PAS_p_ and SR by perceived good stress recovery (REC) was confirmed for SR_G_ (est. mediation path: −0.07, 97.5% CI [−0.13, −0.03]) as well as for SR_S_ (est. mediation path: −0.08, 97.5% CI [−0.12, −0.05]). Mediation in the absence of a direct effect is unusual but statistically plausible, as a significant direct effect is not necessary to interpret the indirect effect (Hayes, 2017). Results of the mediation analyses are depicted in Supplementary Figure S6.

### Exploratory combined multi-variable analyses (regularized regression)

To identify the relatively strongest RFs for both general and specific SR scores, we conducted elastic net analyses for both outcome variables. By cross-validation we arrived at optimal alpha values of α = 0.03 for SR_G_ and α = 0.36 for SR_S_, reflecting greater similarity to ridge than lasso regression. We chose α = 0.03 for both SR scores for reasons of comparability within this sample and to previous studies that relied only on SR_G_. Results for SR_S_ with the alpha value determined by cross-validation are shown in Supplementary Table S11.

**Figure 2:**
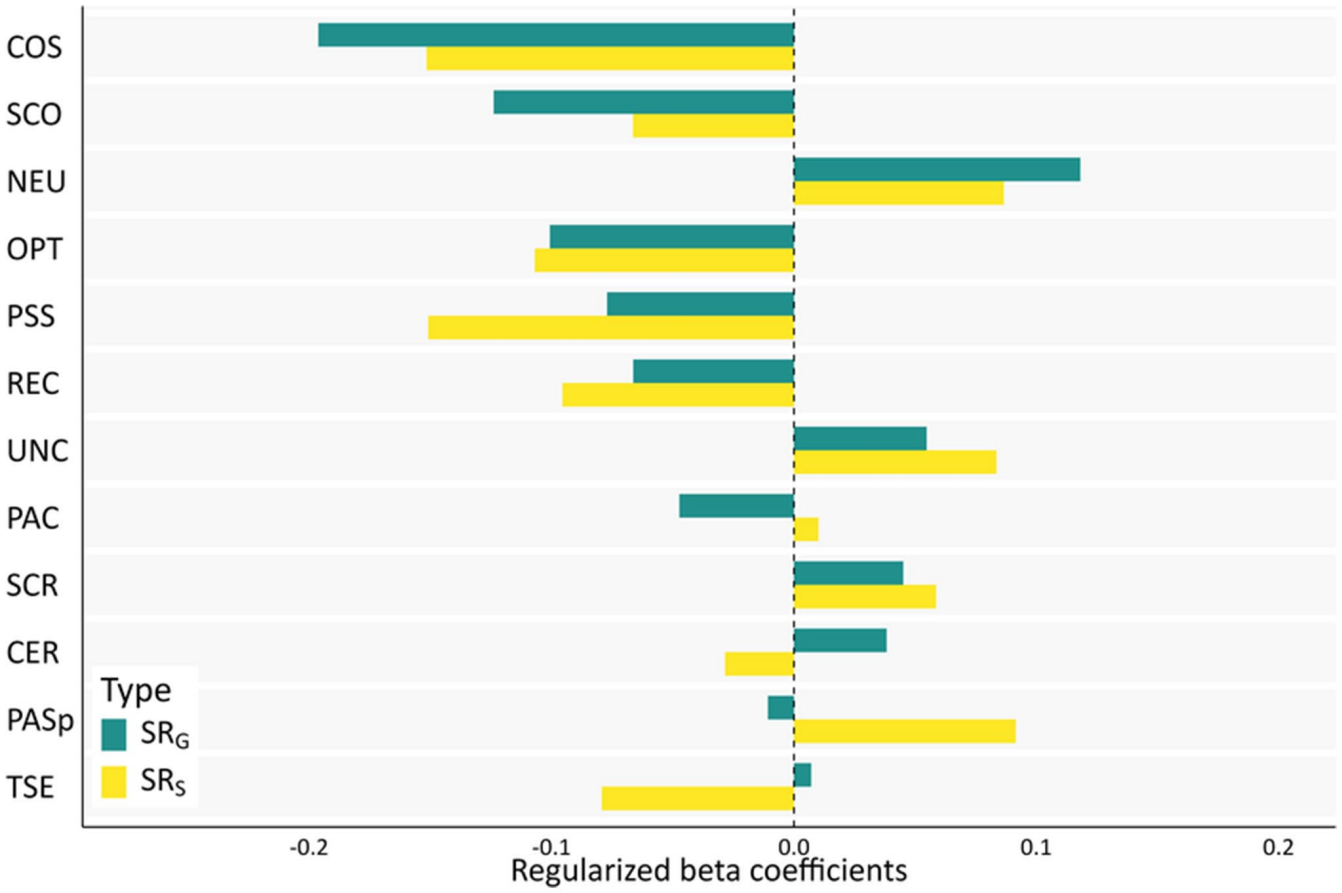
Combined multi-variable analysis (regularized regression) of relative associations between resilience and risk factors and stressor reactivity. Elastic net analysis was conducted separately for both general (SR_G_) and profession-specific (SR_S_) stressor reactivity at an alpha value of 𝛼=0.03, reflecting greater similarity to ridge than lasso regression. Optimal lambda values (λ + 1 SE) were λ = 0.05 for general and λ = 0.1 for profession-specific stressor reactivity. Abbreviations: COS, compassion satisfaction; NEU, neuroticism; OPT, optimism; PSS, perceived social support; PAC, positive appraisal of the Corona crisis; REC, perceived good stress recovery; UNC, uncertainty about mental states; CER, certainty about mental states; SCR, self-criticism; PASp, perceived positive appraisal style – process-focused; TSE, self-efficacy as a therapist.

Regularized beta coefficients for both SR scores at the respective optimal λ values are depicted in Figure 2 and exact values are found in Supplementary Table S11. For all RFs, inclusion frequencies were higher than 95%, indicating high stability in RF selection. For SR_G_, at the optimal λ value of 0.05, the strongest RFs were (in descending order) COS, self-compassion (SCO), neuroticism (NEU), optimism (OPT), and PSS. For SR_S_, the strongest RFs at λ = 0.1 were COS, PSS, OPT, perceived good stress recovery (REC) and PAS_p_. Contradictory to the multiple regression results, PAS_p_ showed a positive association with SR_S_.

## Discussion

In the face of the COVID-19 pandemic, particular focus has been on supporting psychological resilience in health-related professions. Health practitioners are simultaneously at increased risk of stress-related mental health overload and essential for the wellbeing of the wider population (Briggs and Munley, 2008; O'Connor et al., 2018; Aafjes-van Doorn et al., 2022). The present study specifically investigated risk and resilience factors (collectively referred to as RFs) in mental health practitioners, a subgroup among health practitioners that is still understudied and at the same time exposed to a unique profession-related burdens and risks in general and specifically during the pandemic.

In a multi-national sample of mental health practitioners collected during the early phase of the pandemic and mostly comprising psychotherapists, we tested various potential RFs. One set, notably including positive appraisal style and related constructs, has been found to be associated with resilience to pandemic-related internalizing mental health problems in the general population (Veer et al., 2021). We investigated whether these factors would also support wellbeing in mental health practitioners and may thus be classified as global, that is, population-independent (Kalisch et al., 2015). We further investigated whether these factors would be not only associated with resilience to common internalizing problems but also with mental health problems typically observed in stressed psychotherapists and clinical psychologists, namely compassion fatigue and secondary traumatic stress. If so, they could be classified as general, that is, protective against a wider variety of symptoms (Kalisch et al., 2015). We included a second set of RFs that had previously been linked to resilience and wellbeing but has attracted less attention in the context of the pandemic and has not been studied in the population of mental health practitioners, like self-compassion (Ehret et al., 2015; Zessin et al., 2015). Another set of potential factors, including mentalizing, compassion satisfaction and therapist’s self-efficacy had been previously related to therapists’ profession and patient outcomes but either not been associated with therapists’ resilience or not been tested for generalizability across different symptoms in this population (Stamm, 2010; Wilkerson and Basco, 2014; Lingiardi et al., 2018).

### General and global resilience and risk factors

All tested RFs showed significant relationships with general stressor reactivity (SR_G_). Associations of the same RFs with profession-specific stressor reactivity (SR_S_) were always comparable, although minor differences in prediction strength rendered process-focused positive appraisal style (PAS_p_), positive appraisal specifically of the Corona crisis and common sense of humanity non-significant. Speaking to the question of the general nature of the factors, associations with both SR_G_ and SR_S_ were largely identical across tested RFs. It is worth noting that the reported general and profession-specific SR scores correlated moderately at *r* = 0.52. This implies that, apart from an expectedly considerable association between these two measures, they also each captured substantial unique variance. The pattern of results did not change when the associations between RFs and SR_S_ were controlled for the influence of SR_G_, which only led to insignificant results for self-kindness and mindfulness. Together these findings suggest that core aspects of resilient responding, facilitated by RFs, are shared across mental health outcomes that are conceptually distinct and measured with different instruments. The presence of significant associations with both types of SR suggests that the protective influence of the factors perceived social support, optimism, perceived good stress recovery, self-compassion, therapist’s self-efficacy, mentalizing and compassion satisfaction is not limited to internalizing mental health problems but also generalizes to mental health problems typically found in psychotherapists (e.g., burnout or secondary traumatic stress). The same applies to the risk factors neuroticism and self-criticism which showed positive associations with both types of SR.

Similarly, the combined regularized regression of all RFs showed that in this sample of mental health practitioners, three of the five strongest predictors, namely compassion satisfaction, perceived social support and optimism, were shared between SR_G_ and SR_S_. We conclude that even when penalizing association strength and accounting for the influence of many other RFs, the strongest RFs show similar relevance for both outcome measures, which further underlines their generalizability to different types of mental health problems in mental health practitioners.

Speaking to the question of the global nature of the factors, the psychosocial RFs perceived social support, self-perceived good stress recovery, self-compassion, as well as the personality traits optimism and neuroticism had previously been found to be associated with resilience to internalizing symptoms in other studies (e.g., Ehret et al., 2015; Zessin et al., 2015; Veer et al., 2021). All of them were also significant in this specific population and thus generalized well. This suggests that, with regard to their stress response, mental health practitioners rely largely on similar factors as the general population. According to Kalisch et al. (2015), these RFs qualify as being “global” due to their relevance not only for different types of adversity but also for different populations.

### Particular resilience and risk factor constructs

Besides the overall pattern of results, several individual RFs are of particular relevance for resilience in general and for therapists’ resilience specifically.

#### Self-compassion

We find significant associations for self-compassion subscales postulated by Neff (2003) with both types of SR, with the exception of common sense of humanity, which was only significantly associated with SR_G_. The self-compassion compound score was the second most influential factor for SR_G_ in the regularized regression. These results show that mental health practitioners with a compassionate attitude toward themselves show lower reactivity to stressors. In recent years, self-compassion has gained popularity mainly for its applicability in psychotherapy and for association with good patient outcomes (Ferrari et al., 2019). We extend this pattern by demonstrating that self-compassion is a resilience-related concept, opening up new paths for fostering resilience through efficient interventions that benefit practitioners as well as patients.

#### Compassion satisfaction

It is worth noting that compassion satisfaction, an RF we selected for its hypothesized relevance for mental health practitioners, exhibited the strongest associations in the regularized regression analyses for both types of SR. In our study, individuals who indicated to be satisfied with their work with clients and to derive pleasure from helping people showed greater resilience to both general and profession-specific mental health problems. Given the relevance of this factor for this population, it might be worth exploring related constructs in other populations to further evaluate whether it qualifies as a global RF.

#### Mentalizing

Mentalizing was captured in terms of the variables certainty and uncertainty about mental states reasoning. Uncertainty exhibited a consistent negative association with both SR_G_ and SR_S_ in the multiple regression analyses as well as in the regularized regression. This detrimental effect of higher uncertainty about mental states is in line with Fonagy et al. (2016), who found a negative association with wellbeing. Certainty about mental states showed an expected negative association with both types of SR in the multiple regression analyses. In the regularized regression analyses with all RFs considered, however, the expected negative association only emerged for SR_S_ and not SR_G_. For our sample of mental health practitioners this indicates an additional beneficial role of certainty about mental states beyond all other RFs only when dealing with work-related mental health problems. While mentalizing has been proven to have a beneficial association with mental health in different contexts before (Luyten et al., 2020; Maerz et al., 2022; Tohme et al., 2022), we here show its beneficial association with SR in mental health practitioners.

#### Therapist’s self-efficacy

Therapist’s self-efficacy showed much stronger associations with SR_S_ than SR_G_ in the multiple regressions and particularly the regularized regression analyses, indicating its specific importance for preventing profession-related mental health problems. Therapist’s self-efficacy builds on skills that are strictly related to the work as a therapist, which aligns with the higher relevance for SR_S_.

### PASTOR and positive appraisal style

Our work is partly based on the Positive Appraisal Style Theory of Resilience (PASTOR). This theory postulates positive appraisal as the final pathway to resilience (Kalisch et al., 2015). By promoting an optimistic evaluation of an outcome and one’s own coping capabilities, a positive appraisal style makes an adequate and not exaggerated reaction to a stressful event possible. Thus, a more positive appraisal style should lead to a less severe stress reaction, as found by Veer et al. (2021) in a sample from the general population. The effect of other RFs should then be mediated by how they affect positive appraisal style. An example of such an effect, again shown by Veer et al. (2021), is the significant mediation of PAS_p_ on the association between perceived social support and general SR.

The mediating effect of perceived good stress recovery on the association between PAS_p_ and low SR_G_ and SR_S_ identified in this study corroborates PASTOR and is in line with results of Veer et al. (2021). The mediation of PAS_p_ on the association between perceived social support and SR could be replicated for SR_G_ but not for SR_S_. This suggests that the claim of a positive appraisal style as a common pathway to resilience may not generalize to this special type of resilience to profession-specific mental health problems of this unique population. This notion is strengthened by the unexpected positive association between PAS_p_ and SR_S_ in the regularized regression analyses. According to these results, when all other RFs are considered in the model, the unique contribution of PAS_p_ is detrimental for resilience to profession-specific symptoms. This result contradicts the multiple regression results for both SR scores as well as earlier findings (Veer et al., 2021; Bögemann et al., 2022). Thus, rather than considering PAS_p_ a specific risk factor for profession-specific mental health problems, a more likely explanation is that PAS_p_ unfolds its beneficial effect for SR_S_ in a ‘concealed’ fashion through multiple other RFs, like optimism or one’s own perception of good stress recovery. These variables form part of the PASTOR construct and are highly correlated with PAS_p_ (See supplementary Figure S3). This may leave only a small non-beneficial fraction as unique contribution of PAS_p_ in joint analysis. Accordingly, the inconclusive results also reflect certain limitations of regularized regression methods when dealing with high multicollinearity. The same pattern of reversed associations in the regularized regression analyses compared to the multiple regressions were also found for positive appraisal specifically of the Corona crisis, as well as for therapist’s self-efficacy in relation to SR_G_. Reverse coefficients for these RFs were close to zero and thus likely reflect uncertainty in capturing the contribution of RF with low relevance to the overall model.

Regarding the general role of positive appraisal style, both multiple regression and combined regularized regression analyses indicate that mental health practitioners do not strongly rely on PAS_p_ when dealing with mental health problems. It is comprehensible that individuals who are professionally dealing with interventions focusing on emotion regulation and self-reflection might develop a more diverse way of coping than the general population. Our results thus suggest certain limitations of PASTOR for applications for population-specific types of adversity. For general mental health problems, however, the results are compatible with the mechanisms postulated by PASTOR.

### Associations between stressor exposure and mental health problems

Stressor exposure was positively associated with both general, internalizing mental health problems and profession-specific mental health problems involving burnout, compassion fatigue and secondary traumatic stress. The relation to general mental health problems was stronger, likely because stress was measured as challenges occurring in daily rather than professional life, which are less likely to contribute to professional mental health problems. Nonetheless, this data suggest that daily stressors also contribute to profession-specific mental health problems and can generally burden individuals across life-domains.

### Limitations

Several limitations arise from the way data were collected. First of all, the cross-sectional study design does not allow for any inferences about causality and would need to be replicated in a longitudinal design, and the retrospective assessment of stressors of the past 2 weeks further introduces the potential for memory biases. In addition, we cannot rule out the presence of self-report bias in our data which could influence the accuracy of our findings. Further, the snowball sampling approach did not allow us to collect a sample with representative characteristics for the population of mental health practitioners. For example, women were overrepresented. This way of sampling comes with the risk of prevalently including participants willing to disclose their own distress or oversampling of those with heightened stressor load. Thus, despite recruitment of individuals with different demographic backgrounds and a wide range of mental health professions in our suitably sized sample, generalizability of our findings to the broader population of mental health practitioners might be limited.

It should further be mentioned that capturing a full account of individuals coping strategies and personal backgrounds was beyond the scope of this study. We included a wide range of RFs known to be influential in the general population as well as additional factors chosen for this particular population. Still, to keep the burden on participants within reasonable limits, not all potentially contributing factors could be considered.

In addition, we did not directly assess resilient responding to professional challenges and stressors. While we can anticipate that all therapists faced the specific challenges of their profession (e.g., being exposed to patients’ mental health problems and suffering, description of traumatic experiences or insufficient supervision), we did not explicitly measure profession specific stressors. Nonetheless, the only moderate correlation of SR_S_ with SR_G_ indicates that it already covers distinct facets of mental health related to profession-specific resilience.

A last limitation is that the instrument used to assess positive appraisal style in the study was created at speed during the first wave of the pandemic for the purpose of high-throughput online surveys (Veer et al., 2021) and has since been replaced by more extensively validated self-report instruments (Petri-Romão et al., 2023). The principled limitations of self-report necessitate the ongoing development of more objective measures for this new construct (*cf.* Veer et al., 2021).

## Conclusion

To ensure wellbeing in the health care profession and high quality of care for patients, it is important to understand RFs for mental health practitioners, particularly those effective during periods of heightened adversity. Our work identifies psychosocial factors associated with fewer general as well as profession-specific mental health problems after accounting for individual exposure to adversity, including optimism, self-compassion and compassion satisfaction. We find that RFs relevant in the general population mostly generalize to psychotherapists, while additional protective factors are related to their stressors at work during the pandemic. The identified protective factors can be leveraged in targeted interventions or trainings to prevent negative consequences in this unique population. Further investigations into mechanisms underlying resilient responding in mental health practitioners should continue to both develop population-specific resilience concepts and widen the general understanding of resilience.

## Data availability statement

The datasets presented in this study can be found in online repositories. The names of the repository/repositories and accession number(s) can be found at: https://osf.io/pc8tr/.

## Ethics statement

The studies involving humans were approved by Ethical Review Board of the Paul Baerwald School of Social Work and Social Welfare. The studies were conducted in accordance with the local legislation and institutional requirements. Written informed consent for participation was not required from the participants or the participants’ legal guardians/next of kin because participants gave their informed consent online and electronically during data collection.

## Author contributions

MZ: Data curation, Formal analysis, Methodology, Software, Validation, Visualization, Writing – original draft, Writing – review & editing. LP: Formal analysis, Methodology, Software, Supervision, Validation, Writing – original draft, Writing – review & editing. DL: Conceptualization, Funding acquisition, Writing – review & editing. PF: Conceptualization, Resources, Writing – review & editing. PRM: Funding acquisition, Resources, Writing – review & editing. NK: Data curation, Investigation, Project administration, Resources, Writing – review & editing. NL: Data curation, Writing – review & editing. AD: Conceptualization, Project administration, Supervision, Writing – review & editing. RK: Funding acquisition, Methodology, Supervision, Writing – review & editing. TN: Conceptualization, Methodology, Supervision, Writing – original draft, Writing – review & editing.

## Footnotes

^1^www.qualtrics.com

^2^https://www.r-project.org/

## Glossary

BCS: behavioral coping style
CER: certainty about mental states
COH: common humanity
COS: compassion satisfaction
CF_BO: ProQoL burnout scale
CF_STS: ProQoL secondary traumatic stress
E: stressor exposure
GHQ-12: general health questionnaire, 12 items version
ISO: isolation
MIN: mindfulness
NEU: neuroticism
OPT: optimism
OVI: over-identification
P: mental health problems
P_G_: general mental health problems
P_S_: profession-related mental health problems
PAC: positive appraisal specifically of the Corona crisis
PAS: positive appraisal style
PAS_p_: process-focused positive appraisal style
PASTOR: positive appraisal style theory of resilience
PCA: principal component analysis
ProQoL: professional quality of life questionnaire
PSS: perceived social support
REC: perceived good stress recovery
RF: resilience factor
SCO: self-compassion
SCR: self-criticism
SJU: self-judgment
SKI: self-kindness
SR: stressor reactivity
SR_G_: general stressor reactivity
SR_S_: profession-specific stressor reactivity
STSS: secondary traumatic stress scale
TSE: self-efficacy as a therapist
UNC: uncertainty about mental states

## References

[ref1] Aafjes-van Doorn K. Békés V. Luo X. Prout T. A. Hoffman L. (2021b). What do therapist defense mechanisms have to do with their experience of professional self-doubt and vicarious trauma during the COVID-19 pandemic? Front. Psychol. 12. doi: 10.3389/fpsyg.2021.647503 PMC8363079 34393887

[ref2] Aafjes-van Doorn K. Békés V. Luo X. Prout T. A. Hoffman L. (2022). Therapists’ resilience and posttraumatic growth during the COVID-19 pandemic. Psychol. Trauma 14, S165–S173. doi: 10.1037/tra0001097 34472944

[ref3] Aafjes-van Doorn K. Békés V. Prout T. A. (2021a). Grappling with our therapeutic relationship and professional self-doubt during COVID-19: will we use video therapy again? Couns. Psychol. Q. 34, 473–484. doi: 10.1080/09515070.2020.1773404

[ref4] Aafjes-van Doorn K. Békés V. Prout T. A. Hoffman L. (2020). Psychotherapists’ vicarious traumatization during the COVID-19 pandemic. Psychol. Trauma 12, S148–S150. doi: 10.1037/tra0000868, PMID: 32478559

[ref5] Al Maqbali M. Al Sinani M. Al-Lenjawi B. (2021). Prevalence of stress, depression, anxiety and sleep disturbance among nurses during the COVID-19 pandemic: a systematic review and meta-analysis. J. Psychosom. Res. 141:110343. doi: 10.1016/j.jpsychores.2020.110343 33360329 PMC7831768

[ref6] Baron R. M. Kenny D. A. (1986). The moderator-mediator variable distinction in social psychological research: conceptual, strategic, and statistical considerations. J. Pers. Soc. Psychol. 51, 1173–1182. doi: 10.1037//0022-3514.51.6.1173, PMID: 3806354

[ref7] Bögemann S. Puhlmann L. Wackerhagen C. Zerban M. Riepenhausen A. Köber G.. (2022). Psychological resilience factors and their association with weekly stressor reactivity during the COVID-19 outbreak in Europe. JMIR Mental Health. 10:e46518. 10.2196/46518 PMC10618882 37847551

[ref8] Bonanno G. A. Romero S. A. Klein S. I. (2015). The temporal elements of psychological resilience: an integrative framework for the study of individuals, families, and communities. Psychol. Inq. 26, 139–169. doi: 10.1080/1047840X.2015.992677

[ref9] Bonferroni C. (1936). Teoria statistica delle classi e calcolo delle probabilita. Pubblicazioni del R Istituto Superiore di Scienze Economiche e Commericiali di Firenze 8, 3–62.

[ref10] Boscarino J. A. Figley C. R. Adams R. E. (2004). Compassion fatigue following the September 11 terrorist attacks: a study of secondary trauma among new York City social workers. Int. J. Emerg. Ment. Health 6, 57–66. PMID: 15298076 PMC2713725

[ref11] Bride B. E. (2013). The secondary traumatic stress scale, DSM5 revision [Unpublished manuscript]. Available at: http://www.srcac.org/wp-content/uploads/2020/07/18_STSS_DSM_5.pdf

[ref12] Bride B. E. Robinson M. M. Yegidis B. Figley C. R. (2004). Development and validation of the secondary traumatic stress scale. Res. Soc. Work. Pract. 14, 27–35. doi: 10.1177/1049731503254106

[ref13] Briggs D. B. Munley P. H. (2008). Therapist stress, coping, career sustaining behavior and the working alliance. Psychol. Rep. 103, 443–454. doi: 10.2466/pr0.103.2.443-454, PMID: 19102469

[ref14] Clemente-Suárez V. J. Martínez-González M. B. Benitez-Agudelo J. C. Navarro-Jiménez E. Beltran-Velasco A. I. Ruisoto P.. (2021). The impact of the COVID-19 pandemic on mental disorders. A critical review. Int. J. Environ. Res. Public Health 18:10041. doi: 10.3390/ijerph181910041, PMID: 34639341 PMC8507604

[ref15] Cohen J. (1988). Statistical power analysis for the behavioral sciences (2nd ed.). UK: Routledge.

[ref16] Cologon J. Schweitzer R. D. King R. Nolte T. (2017). Therapist reflective functioning, therapist attachment style and therapist effectiveness. Adm. Policy Ment. Health Ment. Health Serv. Res. 44, 614–625. doi: 10.1007/s10488-017-0790-5, PMID: 28132188

[ref17] Crocamo C. Bachi B. Calabrese A. Callovini T. Cavaleri D. Cioni R. M.. (2021). Some of us are most at risk: systematic review and meta-analysis of correlates of depressive symptoms among healthcare workers during the SARS-CoV-2 outbreak. Neurosci. Biobehav. Rev. 131, 912–922. doi: 10.1016/j.neubiorev.2021.10.010, PMID: 34655656 PMC8513395

[ref18] Culver L. M. McKinney B. L. Paradise L. V. (2011). Mental health professionals' experiences of vicarious traumatization in post–hurricane Katrina new Orleans. J. Loss Trauma 16, 33–42. doi: 10.1080/15325024.2010.519279

[ref19] De Brier N. Stroobants S. Vandekerckhove P. De Buck E. (2020). Factors affecting mental health of health care workers during coronavirus disease outbreaks (SARS, MERS & COVID-19): a rapid systematic review. PLoS One 15:e0244052. doi: 10.1371/journal.pone.0244052 33320910 PMC7737991

[ref20] Ehret A. M. Joormann J. Berking M. (2015). Examining risk and resilience factors for depression: the role of self-criticism and self-compassion. Cognit. Emot. 29, 1496–1504. doi: 10.1080/02699931.2014.992394, PMID: 25517734

[ref21] Ferrari M. Hunt C. Harrysunker A. Abbott M. J. Beath A. P. Einstein D. A. (2019). Self-compassion interventions and psychosocial outcomes: a meta-analysis of RCTs. Mindfulness 10, 1455–1473. doi: 10.1007/s12671-019-01134-6

[ref22] Fonagy P. Luyten P. Moulton-Perkins A. Lee Y. W. Warren F. Howard S.. (2016). Development and validation of a self-report measure of mentalizing: the reflective functioning questionnaire. PLoS One 11:e0158678. doi: 10.1371/journal.pone.0158678, PMID: 27392018 PMC4938585

[ref23] Friedman J. Hastie T. Tibshirani R. (2010). Regularization paths for generalized linear models via coordinate descent. J. Stat. Softw. 33, 1–22. doi: 10.18637/jss.v033.i01, PMID: 20808728 PMC2929880

[ref24] Goldberg D. P. Gater R. Sartorius N. Ustun T. B. Piccinelli M. Gureje O.. (1997). The validity of two versions of the GHQ in the WHO study of mental illness in general health care. Psychol. Med. 27, 191–197. doi: 10.1017/s0033291796004242, PMID: 9122299

[ref25] Hastie T. Tibshirani R. Wainwright M. (2015). Statistical learning with sparsity: The lasso and generalizations. UK: Chapman & Hall/CRC Press, 84. 156–157.

[ref26] Hayes A. F. (2017). Introduction to mediation, moderation, and conditional process analysis: A regression-based approach. US: The Guilford Press.

[ref27] Heinonen E. Nissen-Lie H. A. (2020). The professional and personal characteristics of effective psychotherapists: a systematic review. Psychother. Res. 30, 417–432. doi: 10.1080/10503307.2019.1620366 31122157

[ref28] Huang Y. L. Fonagy P. Feigenbaum J. Montague P. R. Nolte T. Mood Disorder Research Consortium (2020). Multidirectional pathways between attachment, mentalizing, and posttraumatic stress symptomatology in the context of childhood trauma. Psychopathology 53, 48–58. doi: 10.1159/000506406, PMID: 32294649 PMC7265765

[ref29] Kalisch R. Baker D. G. Basten U. Boks M. P. Bonanno G. A. Brummelman E.. (2017). The resilience framework as a strategy to combat stress-related disorders. Nat. Hum. Behav. 1, 784–790. doi: 10.1038/s41562-017-0200-8, PMID: 31024125

[ref30] Kalisch R. Köber G. Binder H. Ahrens K. F. Basten U. Chmitorz A.. (2021). The frequent stressor and mental health monitoring-paradigm: a proposal for the operationalization and measurement of resilience and the identification of resilience processes in longitudinal observational studies. Front. Psychol. 12:710493. doi: 10.3389/fpsyg.2021.710493, PMID: 34539510 PMC8444985

[ref31] Kalisch R. Müller M. B. Tüscher O. (2015). A conceptual framework for the neurobiological study of resilience. Behav. Brain Sci. 38:e92. doi: 10.1017/S0140525X1400082X, PMID: 25158686

[ref32] Klasen J. Nolte T. Moeller H. Taubner S. (2019). Adverse childhood experiences, attachment representations and mentalizing capacity of psychotherapists in training. Z. Psychosom. Med. Psychother. 65, 353–371. doi: 10.13109/zptm.2019.65.4.353 31801442

[ref33] Lassri D. Gewirtz-Meydan A. Nolte T. (2022). Where there is Stress, there is Resilience: The Importance of Child Abuse and Neglect in Latent Profiles of Risk and Resilience During the COVID-19 Pandemic- a Transdiagnostic Perspective. (under review).

[ref34] Lingiardi V. Muzi L. Tanzilli A. Carone N. (2018). Do therapists' subjective variables impact on psychodynamic psychotherapy outcomes? A systematic literature review. Clin. Psychol. Psychother. 25, 85–101. doi: 10.1002/cpp.2131, PMID: 28873269

[ref35] Luther L. Gearhart T. Fukui S. Morse G. Rollins A. L. Salyers M. P. (2017). Working overtime in community mental health: associations with clinician burnout and perceived quality of care. Psychiatr. Rehabil. J. 40, 252–259. doi: 10.1037/prj0000234, PMID: 27786520 PMC5574255

[ref36] Lutz W. Leon S. C. Martinovich Z. Lyons J. S. Stiles W. B. (2007). Therapist effects in outpatient psychotherapy: a three-level growth curve approach. J. Couns. Psychol. 54, 32–39. doi: 10.1037/0022-0167.54.1.32

[ref37] Luyten P. Campbell C. Allison E. Fonagy P. (2020). The mentalizing approach to psychopathology: state of the art and future directions. Annu. Rev. Clin. Psychol. 16, 297–325. doi: 10.1146/annurev-clinpsy-071919-015355 32023093

[ref38] Maerz J. Buchheim A. Rabl L. Riedl D. Viviani R. Labek K. (2022). The interplay of criterion a of the alternative model for personality disorders, mentalization and resilience during the COVID-19 pandemic. Front. Psychol. 13:928540. doi: 10.3389/fpsyg.2022.928540 35959052 PMC9358045

[ref39] Manchia M. Gathier A. W. Yapici-Eser H. Schmidt M. V. de Quervain D. Van Amelsvoort T.. (2022). The impact of the prolonged COVID-19 pandemic on stress resilience and mental health: a critical review across waves. Eur. Neuropsychopharmacol. 55, 22–83. doi: 10.1016/j.euroneuro.2021.10.864, PMID: 34818601 PMC8554139

[ref40] Mittal M. Morgan A. A. Du J. Jiang J. Boekeloo B. Fish J. N. (2023). “Each week feels like a mountain”: the impact of COVID-19 on mental health providers’ well-being and clinical work. Prof. Psychol. Res. Pract. 54, 103–113. doi: 10.1037/pro0000501 PMC10228181 37261211

[ref41] Neff K. D. (2003). The development and validation of a scale to measure self-compassion. Self Identity 2, 223–250. doi: 10.1080/15298860309027

[ref42] O'Connor K. Muller Neff D. Pitman S. (2018). Burnout in mental health professionals: a systematic review and meta-analysis of prevalence and determinants. Eur. Psychiatry 53, 74–99. doi: 10.1016/j.eurpsy.2018.06.003 29957371

[ref43] Pappa S. Ntella V. Giannakas T. Giannakoulis V. G. Papoutsi E. Katsaounou P. (2020). Prevalence of depression, anxiety, and insomnia among healthcare workers during the COVID-19 pandemic: a systematic review and meta-analysis. Brain Behav. Immun. 88, 901–907. doi: 10.1016/j.bbi.2020.05.026, PMID: 32437915 PMC7206431

[ref44] Petri-Romão P. Engen H. Rupanova A. Puhlmann L. Zerban M. Neumann R. J.. (2023). Self-report assessment of positive appraisal style (PAS): Development of a process-focused and a content-focused questionnaire for use in mental health and resilience research. PsyArXiv. doi: 10.31234/osf.io/fpw94 PMC10836662 38306328

[ref45] Probst T. Humer E. Stippl P. Pieh C. (2020). Being a psychotherapist in times of the novel coronavirus disease: stress-level, job anxiety, and fear of coronavirus disease infection in more than 1,500 psychotherapists in Austria. Front. Psychol. 11:559100. doi: 10.3389/fpsyg.2020.559100, PMID: 33132965 PMC7550677

[ref46] Riepenhausen A. Veer I. M. Wackerhagen C. Reppmann Z. C. Köber G. Ayuso-Mateos J. L.. (2022). Coping with COVID: risk and resilience factors for mental health in a German representative panel study. Psychol. Med. 53, 3897–3907. doi: 10.1017/S0033291722000563, PMID: 35301966 PMC8943230

[ref47] Robinson E. Sutin A. R. Daly M. Jones A. (2022). A systematic review and meta-analysis of longitudinal cohort studies comparing mental health before versus during the COVID-19 pandemic in 2020. J. Affect. Disord. 296, 567–576. doi: 10.1016/j.jad.2021.09.098, PMID: 34600966 PMC8578001

[ref48] Rogoff S. Moulton-Perkins A. Warren F. Nolte T. Fonagy P. (2021). 'Rich' and 'poor' in mentalizing: do expert mentalizers exist? PLoS One 16:e0259030. doi: 10.1371/journal.pone.0259030 34695171 PMC8544847

[ref49] Rudich Z. Lerman S. F. Gurevich B. Weksler N. Shahar G. (2008). Patients' self-criticism is a stronger predictor of physician's evaluation of prognosis than pain diagnosis or severity in chronic pain patients. J. Pain 9, 210–216. doi: 10.1016/j.jpain.2007.10.013 18055267

[ref50] Schäfer S. K. Kunzler A. M. Kalisch R. Tüscher O. Lieb K. (2022). Trajectories of resilience and mental distress to global major disruptions. Trends Cogn. Sci. 26, 1171–1189. doi: 10.1016/j.tics.2022.09.017, PMID: 36302711 PMC9595401

[ref51] Simionato G. K. Simpson S. (2018). Personal risk factors associated with burnout among psychotherapists: a systematic review of the literature. J. Clin. Psychol. 74, 1431–1456. doi: 10.1002/jclp.22615, PMID: 29574725

[ref52] Stamm B. H. (2010). The concise ProQOL manual. Pocatello. Available at: http://ProQOL.org/uploads/ProQOL_Concise_2ndEd_12-2010.pdf

[ref53] Tohme P. Grey I. El-Tawil M. T. El Maouch M. Abi-Habib R. (2022). Prevalence and correlates of mental health difficulties following the Beirut port explosion: the roles of mentalizing and resilience. Psychol. Trauma Theory Res. Pract. Policy. doi: 10.1037/tra0001328, PMID: 35878088

[ref54] Van Buuren S. (2018). Flexible imputation of missing data, Second Edition. UK: Chapman and Hall/CRC.

[ref55] Van Buuren S. Groothuis-Oudshoorn K. (2011). Mice: multivariate imputation by chained equations in R. J. Stat. Softw. 45, 1–67. doi: 10.18637/jss.v045.i03

[ref56] Van Hoy A. Rzeszutek M. (2023). Trajectories of burnout and psychological well-being among psychotherapists during the Covid-19 pandemic: results of a 1-year prospective study. Stress. Health10.1002/smi.3230 36698253

[ref57] Van Hoy A. Rzeszutek M. Pięta M. Mestre J. M. Rodríguez-Mora Á. Midgley N.. (2022). Burnout among psychotherapists: a cross-cultural value survey among 12 European countries during the coronavirus disease pandemic. Sci. Rep. 12:13527. doi: 10.1038/s41598-022-17669-z, PMID: 35941352 PMC9358385

[ref58] Veer I. M. Riepenhausen A. Zerban M. Wackerhagen C. Puhlmann L. M. C. Engen H.. (2021). Psycho-social factors associated with mental resilience in the Corona lockdown. Transl. Psychiatry 11:67. doi: 10.1038/s41398-020-01150-4, PMID: 33479211 PMC7817958

[ref59] Wakelin K. E. Perman G. Simonds L. M. (2022). Effectiveness of self-compassion-related interventions for reducing self-criticism: a systematic review and meta-analysis. Clin. Psychol. Psychother. 29, 1–25. doi: 10.1002/cpp.2586, PMID: 33749936

[ref60] Wampold B. E. Owen J. (2021). “Therapist effects: history, methods, magnitude, and characteristics of effective therapists” in Bergin and Garfield's handbook of psychotherapy and behavior change: 50th anniversary edition. eds. Barkham M. Lutz W. Castonguay L. G. (US: John Wiley & Sons, Inc), 297–326.

[ref61] Werner A. M. Tibubos A. N. Rohrmann S. Reiss N. (2019). The clinical trait self-criticism and its relation to psychopathology: a systematic review–update. J. Affect. Disord. 246, 530–547. doi: 10.1016/j.jad.2018.12.069, PMID: 30599378

[ref62] Wilkerson A. Basco M. R. (2014). Therapists' self-efficacy for CBT dissemination: is supervision the key? J. Psychol. Psychotherapy 4, 1–6. doi: 10.2196/preprints.50228

[ref63] Zerban M. Puhlmann L. M. C. Lassri D. Fonagy P. Montague P. R. Kiselnikova N.. (2023). What helps the helpers? Resilience and risk factors for general and profession-specific mental health problems in psychotherapists during the COVID-19 pandemic JMIR Preprints. 10.3389/fpsyg.2023.1272199 PMC10757941 38164261

[ref64] Zessin U. Dickhäuser O. Garbade S. (2015). The relationship between self-compassion and well-being: a meta-analysis. Appl. Psychol. Health Well Being 7, 340–364. doi: 10.1111/aphw.12051 26311196

