# Supplementary material for: What helps the helpers? Resilience and risk factors for general and profession-specific mental health problems in psychotherapists during the COVID-19 pandemic
Source: Front Psychol. 2023 Dec 18;14:1272199. doi: 10.3389/fpsyg.2023.1272199 (PMC10757941; doi:10.3389/fpsyg.2023.1272199)
Supplement: Supplementary file 1 [file Data_Sheet_1.pdf]

Supplementary Material

For the interested reader, the supplementary material provides additional information about methods, main analyses as well as additional analyses that were beyond the scope of the main text.

Content

Supplementary Sections

Section S1: Missing data imputation.

Section S2: Regularized regression.

Supplementary Figures

Figure S1: Box-whisker plots for observed and imputed data.

Figure S2: Missing data pattern in the final dataset dataset (N=569) and their frequency.

Figure S3: Correlation matrix for resilience factors, stressor reactivity (SR) and outcome related measures.

Figure S4: Associations between hypothesized resilience and risk factors (RFs) and stressor. reactivity (SR) in unimputed dataset.

Figure S5. Distribution of residuals of resilience factors.

Figure S6: Results of mediation analysis.

Figure S7: Associations between hypothesized resilience and risk factors (RFs) and stressor reactivity (SRS) with and without controlling for general stressor reactivity (SRG).

Supplementary Tables

Table S1: Overview of employed instruments and variables.

Table S2: Sample characteristics.

Table S3: Results of the Principal Component Analysis including all ProQoL Scales and STSS.

Table S4: Results of likelihood-ratio test for covariate inclusion.

Table S5-S7: Results of multiple regression analyses with general SR score (SR_G).

Table S8-S10: Results of multiple regression analyses with general SR score (SR_S).

Table S11: Regularized beta coefficients of resilience and risk factors (RFs) and covariates.

Section S1: Missing data imputation

An insignificant result of Little’s test (Little, 1988) supported that data was missing completely at random (MCAR). Meeting the criteria of MCAR would allow for simple imputation methods, like mean imputation. However, since this test is not able to correctly identify MCAR under all circumstances (Jakobsen et al., 2017), the more sophisticated method of multiple imputation (MI) was chosen, which is also working under the broader assumption of data missing at random (van Buuren, 2018). Presence of connected missing data patterns with multivariate missingness made MI possible. MI accounts for uncertainties in the imputation process by choosing a set of probable candidates for a missing data point and then imputing multiple datasets by drawing from this set of values. The number of imputations was set to m=20. Box-whisker plots of observed and imputed data for the individual variables is depicted in Supplementary Figure S1.

Section S2: Regularized regression

In elastic net, the L_1-Norm of the coefficients is penalized with a parameter λ in a way that biases the beta coefficients towards zero, including setting them to zero, thus performing regularization and variable selection (Hastie et al., 2015). The elastic net is therefore used for identifying important predictors in multi-variate settings and integrating them into a robust model. Optimal combination of lasso and ridge elements was determined via cross-validation and is indicated by the term alpha, which is set between 0 (i.e., ridge regression) and 1 (i.e., LASSO regression). We subsequently selected λ based on cross-validation to identify a subset of variables that is particularly suited for predicting SR. To minimize risk of overfitting and maximize generalizability, optimal λ was defined as the λ that minimizes cross-validation error +1 SE, a criterion designed to select the simplest model whose accuracy is comparable to the best model (Friedman et al., 2010; Krstajic et al., 2014).

Figure S1

Box-whisker plots for observed and imputed data

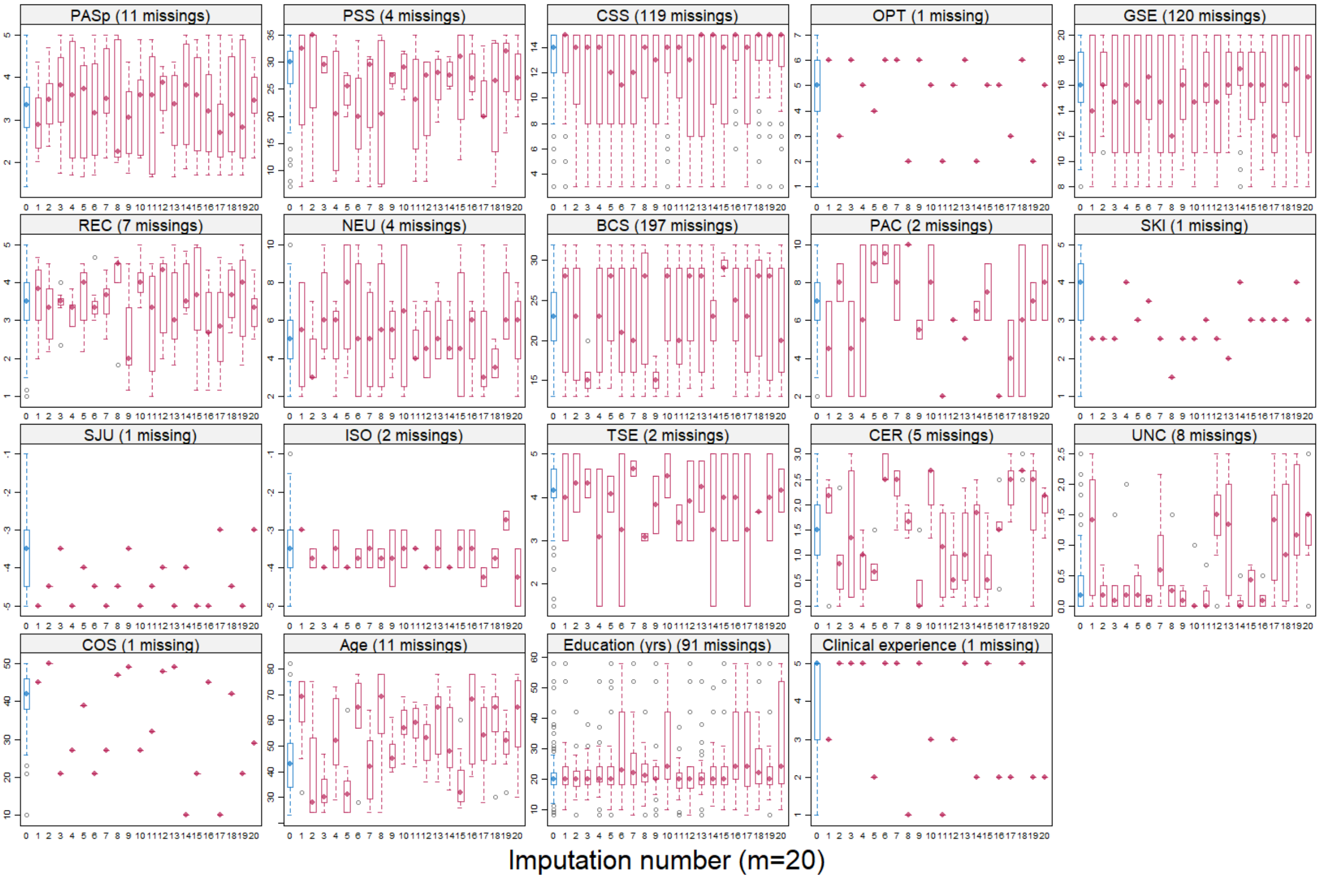

Note. Plots are shown for all numeric variables. Blue graphs show density distribution of observed data, red graphs for all m=20 imputations. Abbreviations: PASp, processed-focused positive appraisal style; PSS, perceived social support; CSS, Corona-related social support; OPT, optimism; GSE, general self-efficacy; REC, perceived good stress recovery; NEU, neuroticism; BCS, behavioural coping style; PAC, positive appraisal of the Corona crisis; SKI, self-kindness; SJU, self-judgment; ISO, isolation; SCR, self-criticism; TSE, self-efficacy as a therapist; CER, certainty about mental states; UNC, uncertainty about mental states; COS, compassion satisfaction.

Figure S2

Missing data patterns in the final dataset (N=569) and their frequency

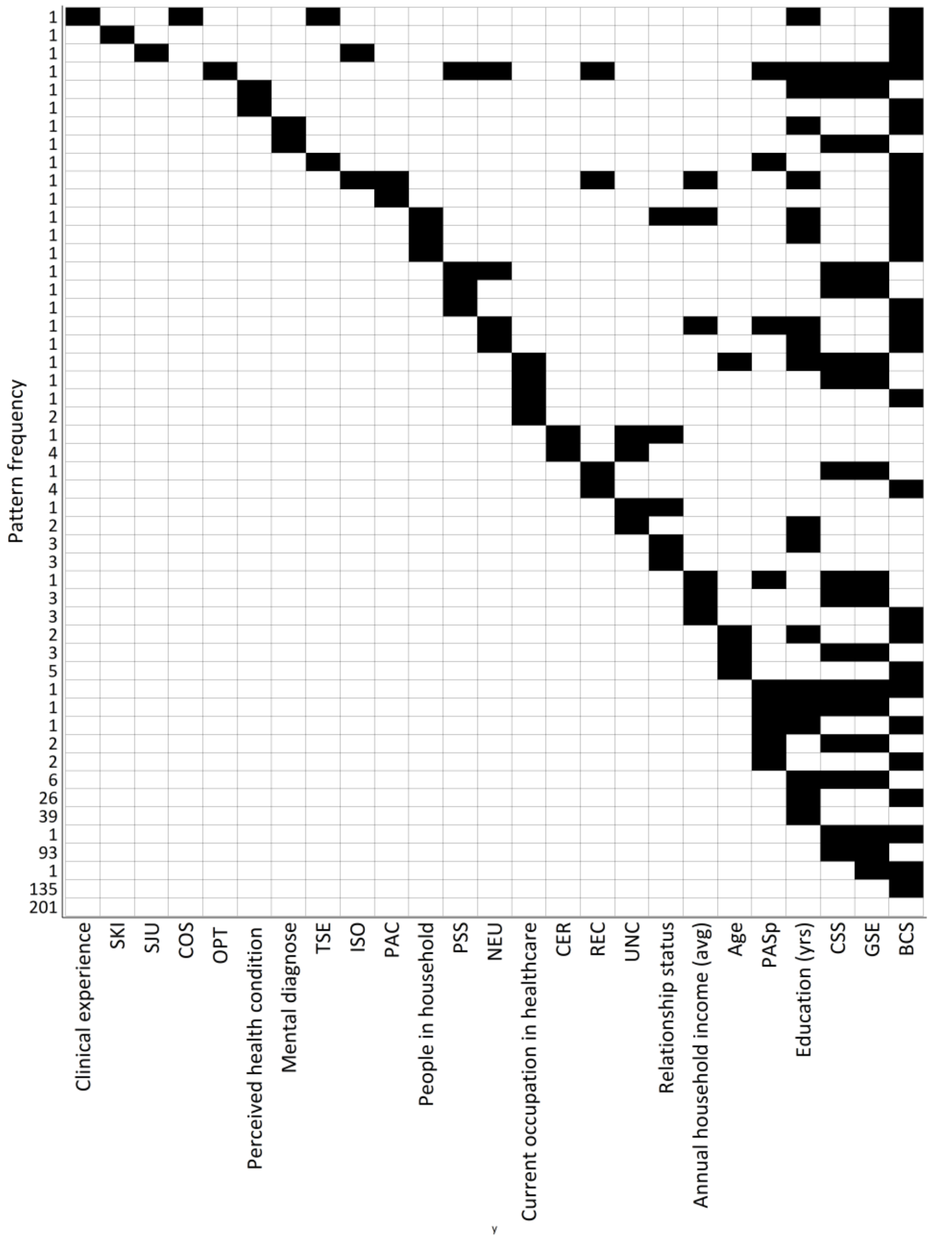

Note. Black tiles indicate missing data points for a given variable, such that the first pattern represents missing data in each of Clinical experience, COS, TSE, Education and BCS (n=1), while the last two patterns show missing data only in BCS (n=135) and no missing data (n=201). Only variables with missing data are listed. Abbreviations: SKI, self-kindness; SJU, self-judgment;

COS, compassion satisfaction; OPT, optimism; TSE, self-efficacy as a therapist; ISO, isolation; PAC, positive appraisal of the Corona crisis; PSS, perceived social support; NEU, neuroticism; CER, certainty about mental states; REC, perceived good stress recovery; UNC, uncertainty about mental states; PASp, processed- focused positive appraisal style; CSS, Corona-related social support; GSE, general self-efficacy; BCS, behavioural coping style.

Figure S3

Correlation matrix for resilience and risk factors, stressor reactivity (SR) and outcome related measures

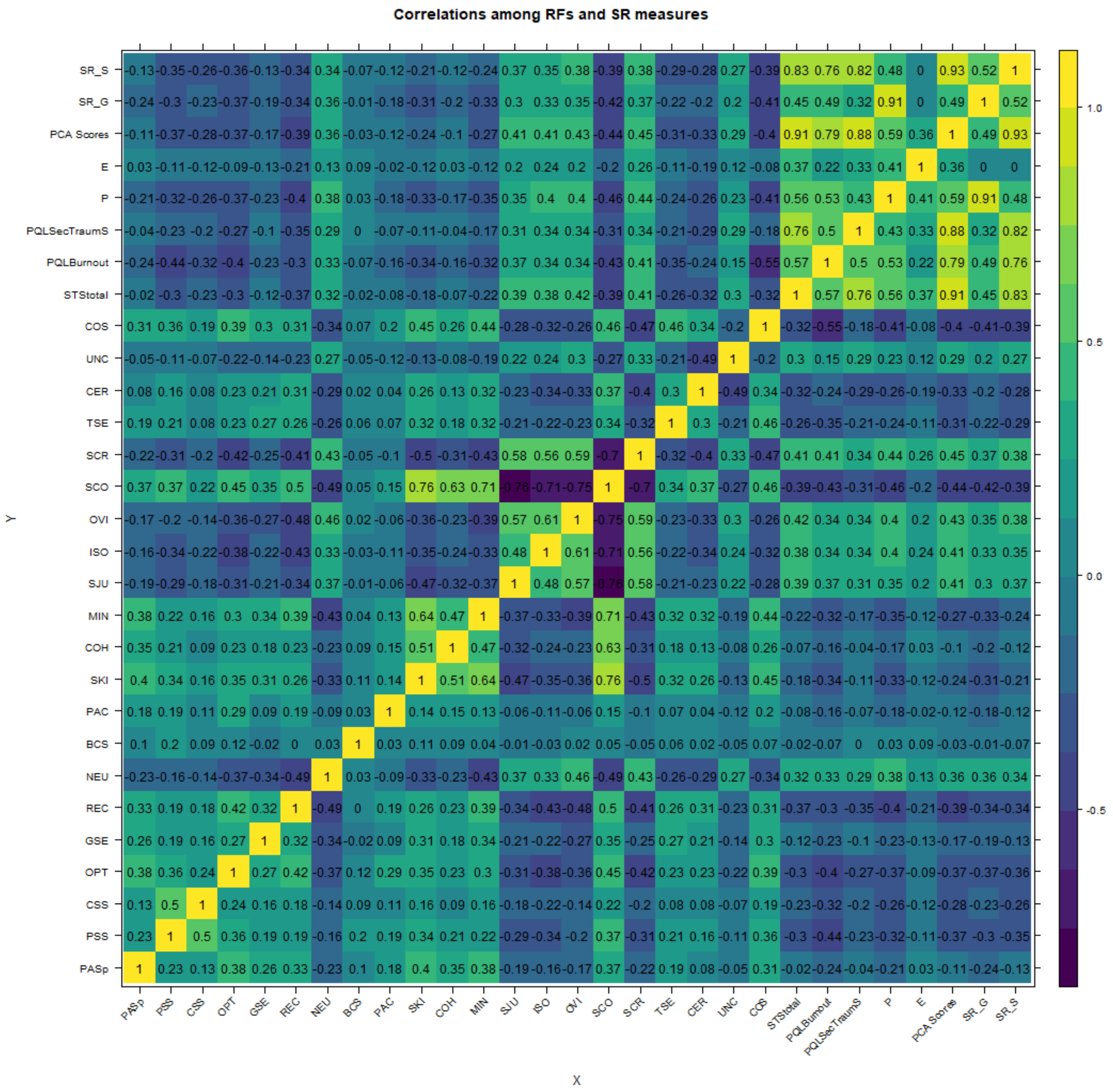

Note. Abbreviations: PASp, processed-focused positive appraisal style; PSS, perceived social support; CSS, Corona-related social support; OPT, optimism; GSE, general self-efficacy; REC,

perceived good stress recovery; NEU, neuroticism; PAC, positive appraisal of the Corona crisis; SKI, self-kindness; COH, common humanity, MIN, mindfulness; SJU, self-judgment; ISO, isolation; OVI, overidentification; SCO, self-compassion; SCR, self-criticism; TSE, self-efficacy as a therapist; CER, certainty about mental states; UNC, uncertainty about mental states; COS, compassion satisfaction; STStotal, sumscore of Secondary Trauma Stress Scale; ProQoL, Professional Quality of LIfe Scale; P, mental health problems (GHQ-12 Sumscore); E, stressor exposure; PCA Scores, PCA scores of first component, see methods section; SR_G, general SR score; SR_S, specific SR Score;

Figure S4

Associations between hypothesized resilience and risk factors (RFs) and stressor reactivity (SR) in unimputed dataset

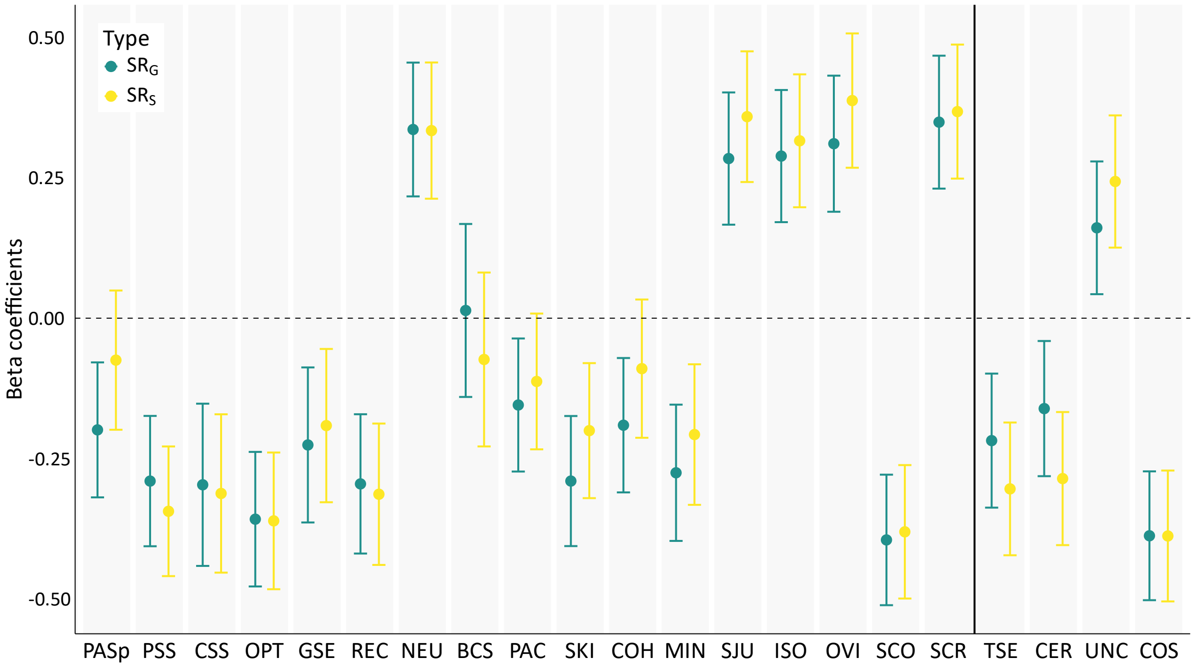

Note. Shown are standardized beta coefficients of RFs predicting SR in multiple regressions, calculated separately for each RF. Coefficients of profession-relevant RFs are placed on the right. Covariates age, gender, current relationship status, people in household and clinical experience in years are included in each model. PASp, process-focused positive appraisal style (n=531); PSS, perceived social support (n=538); CSS, Corona-related social support (n=429); OPT, optimism (n=541); GSE, general self-efficacy (n=428); REC, perceived good stress recovery (535); NEU, neuroticism (n=538); PAC, positive appraisal of the Corona crisis (n=540); SKI, self-kindness (n=541); COH, common humanity (n=542); MIN, mindfulness (n=542); SJU, self-judgment (n=541); ISO, isolation (n=540); OVI, overidentification (n=542); SCO, self-compassion (n=542); SCR, self-criticism (n=542); TSE, self-efficacy as a therapist (n=541); CER, certainty about

mental states (n=538); UNC, uncertainty about mental states (n=539); COS, compassion satisfaction (n=542). Error bars depict 99% Confidence intervals.

Figure S5

Distribution of residuals of resilience and risk factors

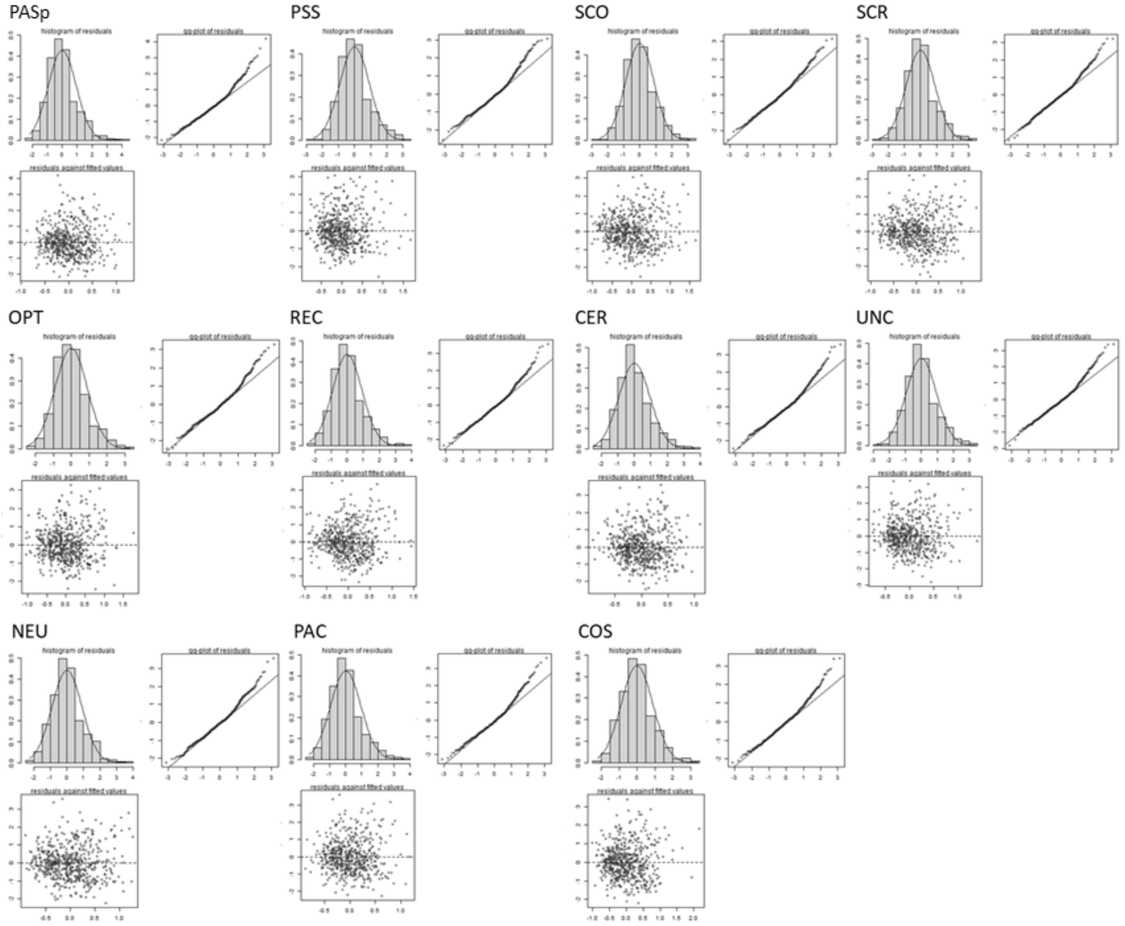

Note. Depicted are histograms and qq-plots of residuals as well as scatterplots plotting residuals against fitted values. Abbreviations: PASp, process-focused positive appraisal style; PSS, perceived social support; OPT, optimism; REC, perceived good stress recovery; NEU, neuroticism; PAC, positive appraisal of the Corona crisis; SCO, self-compassion; SCR, self-criticism; TSE, self-efficacy as a therapist; CER, certainty about mental states; UNC, uncertainty about mental states; COS, compassion satisfaction.

Figure S6

Results of mediation analyses

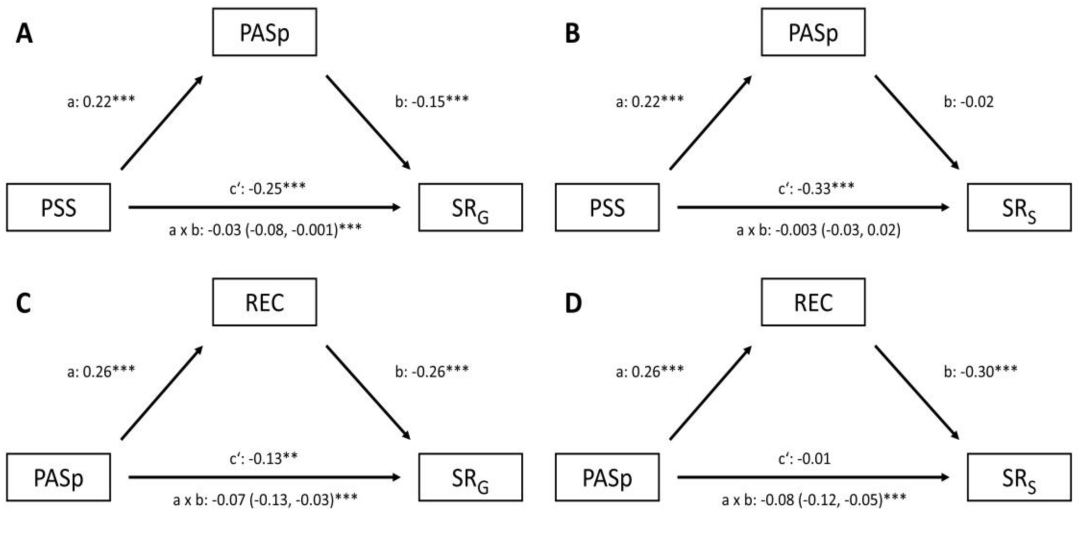

Note. Mediation analyses testing if (A and B) the positive association of perceived social support (PSS) with stressor reactivity (SR) is mediated by positive appraisal style (PASp) and (2C and 2D) if the positive association of PASp on SR is mediated by perceived good stress recovery (REC). Shown are β of all paths. Indirect path a × b: β with 99% CI. **p<.01 ***p < .001. SR_G, general stressor reactivity; SR_S, profession-specific stressor reactivity.

Figure S7

Associations between hypothesized resilience and risk factors (RFs) and stressor reactivity (SR_S) with and without controlling for general stressor reactivity (SR_G)

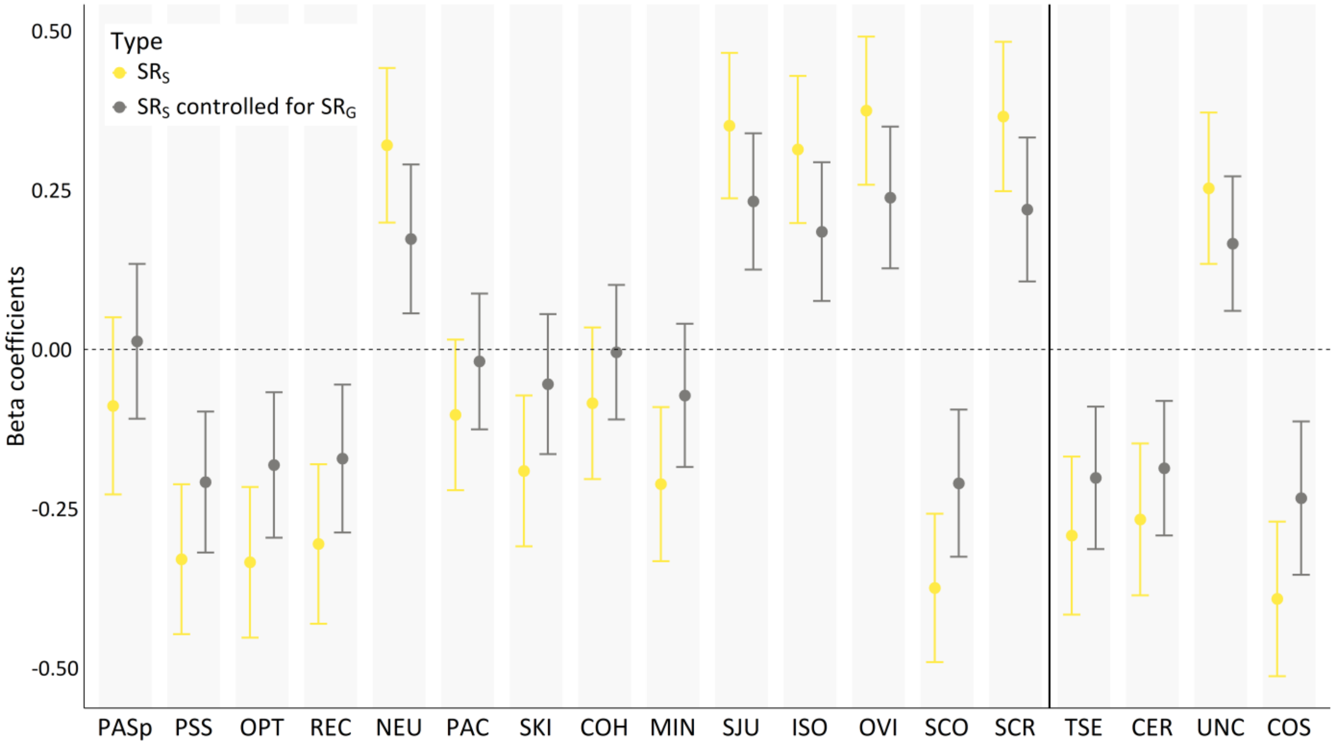

Note. Shown are standardized beta coefficients of RFs predicting SR in multiple regressions, calculated separately for each RF. Coefficients of profession-relevant RFs are placed on the right. Covariates age, gender, current relationship status, people in household and clinical experience in years are included in each model. PASp, process-focused positive appraisal style; PSS, perceived social support; CSS, Corona-related social support; OPT, optimism; GSE, general self-efficacy; REC, perceived good stress recovery; NEU, neuroticism; PAC, positive appraisal of the Corona crisis; SKI, self-kindness; COH, common humanity; MIN, mindfulness; SJU, self-judgment; ISO, isolation; OVI, overidentification; SCO, self-compassion; SCR, self-criticism; TSE, self-efficacy as a therapist; CER, certainty about mental states; UNC, uncertainty about mental states; COS, compassion satisfaction. Error bars depict 99% Confidence intervals.

Table S1

Overview of employed instruments and variables

Variable name (Abbreviation)	Instrument description
**Stressor exposure (E)**	Combined occurrence and severity rating of 11 general (e.g., negative political events, experiencing mental health problems, burdensome experiences at home or with one's family, etc.) and 29 COVID-19-specific pandemic (e.g., being at increased risk for an infection, loss of social contact, having COVID-19 symptoms, etc.) stressors. Participants report whether the events are currently occurring or have occurred in the past two weeks and as how straining they were experienced on a 5-point Likert scale (Veer et a., 2021). Computed as weighted sum score.
**Mental health problems (P)**	
P_G	General Health Questionnaire (GHQ-12). Symptoms of anxiety, depression, insomnia, social problems as well as somatic symptoms, 12 items scoring on a 4-point Likert scale, sum score (Goldberg et al., 1997).
P_S (components)	*Secondary Trauma Stress Scale (STSS):* Self-report measure of secondary trauma stress, 21 items (*DSM-5 Revision version*; Bride et al., 2013), that corresponds with the DSM-5 definition. Comprises the four subscales assessing symptoms of intrusion, avoidance, negative cognitions & mood, and arousal (Bride et al., 2004). 5-point Likert scale.<br>*Professional Quality of Life Questionnaire (ProQoL):*<br>The subscales compassion fatigue: secondary trauma (CF_ST) and compassion fatigue: burnout (CS_BO) were used as symptom measures (*Version 5;* Stamm, 2010). The ProQoL is a commonly used measure of the positive and negative outcomes of working with individuals who have experienced extremely stressful events. It is a reliable and valid measure in terms of construct validity and has good internal consistency, with alpha reliability results ranging from 0.75 to 0.88 (Stamm, 2010). The structure of the ProQoL emerges robustly in factor analyses (Stamm, 2010). 5-point Likert scale.
**Resilience and risk factors (RFs) identified in other populations**	

Perceived Positive Appraisal Style - process-focused (PAS_p)	Assessment of positive appraisal style focusing on cognitive processes that generate positive appraisal contents in stressful situations. Referred to as PAS in Veer et al. (2021). PAS_p is derived from a selection of items from the brief COPE (Carver, 1997), the CERQ-short (Garnefski & Kraaij, 2006), reflecting acceptance, positive reappraisal, putting into perspective, as well as two additionally formulated items on distancing (detachment). It is computed as a composite score by taking the average of the z-normalized scores of the COPE items (scoring 1 to 4), the CERQ items (scoring 1 to 5), and the self-generated items (scoring 1 to 5). This work is based on a preliminary version of the construct (Petri-Romão et al., 2023)
Positive Appraisal specifically of the COVID-19 pandemic (PAC)	Assessment of the appraisal of the consequences of the COVID-19 pandemic on one's own life as well as society. The 2 items were self-generated by Veer et al. (2021). 5-point Likert scale. Computed as sum score.
Neuroticism (NEU)	Neuroticism scale of the NEO Five Factor Inventory (NEO-FFI), 12 items (Rammstedt and John, 2007). 5-point Likert scale. Computed as sum score.
Perceived good stress recovery (REC)	Ability to recover from stressful events assessed by the 6-item Brief Resilience Scale (BRS; Smith et al., 2008). 5-point Likert scale. Computed as sum score.
Optimism (OPT)	Single item scoring 1 (not at all optimistic) to 7 (very optimistic). Self-generated for Veer et al., 2021; Bögemann et al., 2022.
Perceived social support and social belonging (PSS)	Assessed with the 7-item short version of F-SozU (Dunkel et al., 2005). 5-point Likert scale. Computed as sum score.
Perceived changes in social support related to the COVID-19 pandemic (CSS)	Assessed with one item about whether social support was perceived as being reduced or increased over the course of the pandemic (Veer et al., 2021). 5-point Likert scale.
Perceived general self-efficacy (GSE)	Assessed with the English version of the ASKU scale (Beierlein, 2012). This short scale consists of 3 items. 5-point Likert scale. Computed as sum score.
Behavioral Coping Style (BCS)	Selection of 8 items of the brief COPE (Carver, 1997), covering behaviourally oriented coping styles (see supplement of Veer et al., 2021, section 2.2.2 for details on development). 4-point Likert scale. Computed as sum score.

Self-criticism (SCR)	Assessed using the DEQ-SC6, a validated 6-item measure of self-criticism (see Rudich et al., 2008) based on the self-criticism subscale drawn from the original 66-item scale of the Depressive Experiences Questionnaire (Blatt et al., 1976). The DEQ-SC6 uses a 7-point Likert scale for each item. Computed as mean score.
Self-compassion (SCO) + subscales (SKI, COH, MIN, SJU, ISO, OVI)	Assessed using the Self-Compassion Scale–Short Form (SCS–SF; Neff, 2003), a 12-item self-report questionnaire assessing traits reflecting self-kindness (SKI), common humanity (COH), and mindfulness (MIN), as well as the negative poles self-judgment (SJU), isolation (ISO), and overidentification (OVI). Subscales reflected by 2 items each and SCO assessed as composite score of all 12 items. 5-point Likert scale. All scores computed as mean scores.
**Profession-relevant resilience and risk factors**	
Mentalizing (CER and UNC)	Assessed by the short 8-item version of Reflective Functioning Questionnaire (RFQ8; Fonagy et al., 2016), a reliable and valid instrument for assessing individuals' self-reported tendencies to consider mental states as relevant to understanding their own and others' behaviour. The RFQ8 yields two subscales: certainty (CER) and uncertainty (UNC) about mental states. The CER subscale reflects an adaptive facet of genuine and effective mentalizing. 7-point Likert scale. Computed as mean score.
Therapist self-efficacy (TSE)	Assessed by 6-item instrument assessing self-evaluations of essential psychotherapeutic abilities (Wilkerson & Basco; 2014). 5-point Likert scale. Computed as mean score.
Compassion Satisfaction (COS)	Assessed by 10 items about the positive feelings arising from the ability to help people. Subscale from ProQoL (Stamm, 2010). 5-point Likert scale. Computed as mean score.
**Covariate candidates**	
Socio-demographic information	Age, gender, questionnaire language, current occupation in health care, average annual household income, current relationship status, people living in household, years of education, clinical experience in years
Health characteristics	Belonging to COVID-19 risk group, perceived health condition compared to others of same age, diagnosis of mental disorder given by doctor

Note. Citations refer only to the original publications and not the validation studies of translated versions. Self-developed questionnaires are available upon request. A full list of all collected demographic items can be found on OSF (https://osf.io/pc8tr) The

short version of the Experience in Close Relationships (ECR-RD12; revised version; Brenk-Franz et al., 2018) was featured in the study questionnaire but had to be excluded from the analyses due to incoherences in the data collection that could not be understood afterwards. The following qualitative items were answered as free text and were not featured in the analyses due to time-demanding pre-processing: “Which obstacles/problems do you have to face during the Corona pandemic?”; “What is it that helps you the most during the Corona pandemic?”; “Is there something else you would like to tell us?”

Table 2

Sample characteristics

	Male (N=81)	Female (N=488)	Overall (N=569)
**Age (years)**			
Mean (SD)	44.74 (12.94)	43.39 (11.66)	43.58 (11.85)
Median [Min, Max]	43.0 [23.0, 82.0]	43.0 [23.0, 78.0]	43.0 [23.0, 82.0]
**Response Language (N)**			
English	43 (53.1 %)	213 (43.6 %)	256 (45.0 %)
Hebrew	10 (12.3 %)	108 (22.1 %)	118 (20.7 %)
Russian	28 (34.6 %)	167 (34.2 %)	195 (34.3 %)
**Education (years)**			
Mean (SD)	21.28 (3.84)	20.50 (5.00)	20.62 (4.85)
Median [Min, Max]	20.0 [9.0, 35.0]	20.0 [8.0, 58.0]	20.0 [8.0, 58.0]
**Clinical experience (years)**			
Mean (SD)	4.12 (1.02)	4.14 (1.10)	4.14 (1.09)
Median [Min, Max]	5.0 [2.0, 5.0]	5.0 [1.0, 5.0]	5.0 [1.0, 5.0]
**Current occupation in health care (N)**			
No	13 (16.0 %)	140 (28.7 %)	153 (26.9 %)
Yes	67 (82.7 %)	344 (70.5 %)	411 (72.2 %)
NA	1 (1.2 %)	4 (0.8 %)	5 (0.9 %)
**Average annual household income (N)**			
€0-€4,999	2 (2.5 %)	20 (4.1 %)	22 (3.9 %)
€5,000-€9,999	12 (14.8 %)	66 (13.5 %)	78 (13.7 %)
€10,000-14,999	10 (12.3 %)	73 (15.0 %)	83 (14.6 %)
€15,000-€24,999	10 (12.3 %)	54 (11.1 %)	64 (11.2 %)
€25,000-€49,999	15 (18.5 %)	99 (20.3 %)	114 (20.0 %)
€50,000-€74,999	14 (17.3 %)	68 13.9 %)	82 (14.4 %)
€75,000-€99,999	5 (6.2 %)	42 (8.6 %)	47 (8.3 %)
€100,000-€124,999	4 (4.9 %)	21 (4.3 %)	25 (4.4 %)
€125,000-€149,999	2 (2.5 %)	11 (2.3 %)	13 (2.3 %)
€150,000-€174,999	0 (0.0 %)	9 (1.8 %)	9 (1.6 %)
€175,000-€200,000	0 (0.0 %)	8 (1.6 %)	8 (1.4 %)
>€200,000	6 (7.4 %)	8 (1.6 %)	14 (2.5 %)
NA	1 (1.2 %)	9 (1.8 %)	10 (1.8 %)
**Current relationship status (N)**			

Married, in a domestic partnership or civil union	50 (61.7 %)	278 (57.0 %)	328 (57.6 %)
In a Relationship	16 (19.8 %)	75 (15.4 %)	91 (16.0 %)
Single	12 (14.8 %)	129 (26.4 %)	141 (24.8 %)
NA	3 (3.7 %)	6 (1.2 %)	9 (1.6 %)
**People living in household (N)**			
1	12 (14.8 %)	61 (12.5 %)	73 (12.8 %)
2	27 (33.3 %)	158 (32.4 %)	185 (32.5 %)
3 to 4	32 (39.5 %)	211 (43.2 %)	243 (42.7 %)
5 to 6	8 (9.9 %)	48 (9.8 %)	56 (9.8 %)
More than 6	1 (1.2 %)	8 (1.6 %)	9 (1.6 %)
NA	1 (1.2 %)	2 (0.4 %)	3 (0.5 %)
**Belonging to COVID-19 risk group (N)**			
Yes	13 (16.0 %)	73 (15.0 %)	86 (15.1 %)
No	67 (82.7 %)	380 (77.9 %)	447 (78.6 %)
Unsure	1 (1.2 %)	35 (7.2 %)	36 (6.3 %)
**Health condition compared to others of same age (N)**			
Less healthy / more frequently ill	4 (4.9 %)	33 (6.8 %)	37 (6.5 %)
Equally healthy / equally frequently ill	33 (40.7 %)	248 (50.8 %)	281 (49.4 %)
Healthier / less frequently ill	30 (37.0 %)	135 (27.7 %)	165 (29.0 %)
A lot healthier / much less frequently ill	13 (16.0 %)	61 (12.5 %)	74 (13.0 %)
Never ill	1 (1.2 %)	9 (1.8 %)	10 (1.8 %)
NA	0 (0.0 %)	2 (0.4 %)	2 (0.4 %)
**Presence of Mental Health Diagnosis (N)**			
No	66 (81.5 %)	397 (81.4 %)	463 (81.4 %)
Yes	15 (18.5 %)	89 (18.2 %)	104 (18.3 %)
NA	0 (0.0 %)	2 (0.4 %)	2 (0.4 %)

Note. All analyses are based on this sample of N=569 participants. SD, standard deviation.

Table S3

Results of the Principal Component Analysis including all ProQoL Scales and STSS

	Components			
	1	2	3	4
**Importance of Components**				
Eigenvalues	2.43	.94	.36	.23
Proportion of variance	.61	.24	.09	.06
Cumulative proportion	.61	.85	.94	1.00
**Component loadings**				
STSS	.56	.33	.28	.71
ProQoL: CF/STS	.51	.50	.16	-.69
ProQoL: CF/Burnout	.54	-.26	-.80	.02
ProQoL: Compassion Satisfaction	-.39	.76	-.50	.15

Note. Abbreviations: STS, Secondary Trauma Stress Scale; CF/STS, Compassion Fatigue/Secondary Traumatic Stress; CF/ Burnout, Compassion Fatigue/Burnout; ProQoL marks scales of the Professional Quality of Life Questionnaire.

Table S4

Results of likelihood-ratio test for covariate inclusion

	p-value		
**Covariate**	**SR_G**	**SR_S**	**Included in models?**
Age	0.045	0.575	yes
Gender	0.040	0.022	yes
Current occupation in health care?	0.362	0.220	no
Average annual household income	0.509	0.519	no
Current relationship status	0.001	0.013	yes
People living in household	0.000	0.006	yes
COVID-19 risk group?	0.724	0.047	yes
Education (years)	0.673	0.391	no
Clinical experience (years)	0.167	0.453	yes
Perceived health condition	0.032	0.056	yes
Mental health diagnosis?	0.003	0.004	yes
Survey language	0.272	0.381	no

Table S5

Associations between hypothesized general resilience and risk factors (RFs) and general stressor reactivity (SR_G), first part

PAS_p	**-0.21 [-0.32, -0.09] ***					
PSS		**-0.28 [-0.39, -0.17] ***				
OPT			**-0.35 [-0.46, -0.25] ***			
REC				**-0.30 [-0.42, -0.19] ***		
NEU					**0.34 [0.23, 0.44] ***	
PAC						**-0.17 [-0.28, -0.07] ***
Age	-0.00 [-0.01, 0.01]	-0.00 [-0.01, 0.01]	-0.00 [-0.01, 0.01]	-0.00 [-0.01, 0.01]	0.00 [-0.01, 0.01]	-0.00 [-0.01, 0.01]
Gender: Female	0.22 [-0.08, 0.52]	0.30 [0.00, 0.59]	0.32 [0.03, 0.61]	0.15 [-0.14, 0.45]	0.07 [-0.22, 0.37]	0.29 [-0.02, 0.59]
Relationship status: Other	0.24 [-0.08, 0.56]	0.21 [-0.11, 0.52]	0.23 [-0.08, 0.53]	0.27 [-0.04, 0.59]	0.31 [0.01, 0.62]	0.27 [-0.05, 0.59]
Relationship status: Single	0.10 [-0.19, 0.39]	0.04 [-0.24, 0.33]	0.02 [-0.25, 0.30]	0.02 [-0.27, 0.30]	0.13 [-0.15, 0.41]	0.07 [-0.22, 0.36]
People in household: 2	-0.28 [-0.67, 0.11]	-0.18 [-0.56, 0.20]	-0.31 [-0.68, 0.06]	-0.29 [-0.67, 0.09]	-0.30 [-0.68, 0.07]	-0.31 [-0.70, 0.08]
People in household: 3-4	**-0.46 [-0.83, -0.09] ***	-0.35 [-0.72, 0.01]	**-0.47 [-0.83, -0.11] ***	**-0.45 [-0.81, -0.08] ***	-0.39 [-0.75, -0.03]	**-0.49 [-0.86, -0.11] ***
People in household: 5-6	-0.46 [-0.94, 0.02]	-0.39 [-0.87, 0.08]	-0.39 [-0.85, 0.07]	-0.44 [-0.91, 0.03]	-0.44 [-0.91, 0.02]	-0.48 [-0.97, 0.00]
People in household: >6	-0.42 [-1.31, 0.48]	-0.62 [-1.49, 0.26]	-0.48 [-1.33, 0.38]	-0.55 [-1.42, 0.32]	-0.44 [-1.30, 0.42]	-0.45 [-1.35, 0.44]
Clinical experience (yrs)	0.02 [-0.10, 0.14]	-0.00 [-0.12, 0.11]	0.02 [-0.10, 0.13]	0.04 [-0.08, 0.16]	0.02 [-0.09, 0.14]	0.02 [-0.10, 0.14]
PH: Equally healthy	-0.23 [-0.68, 0.23]	-0.31 [-0.75, 0.13]	-0.24 [-0.67, 0.19]	-0.19 [-0.64, 0.25]	-0.19 [-0.63, 0.25]	-0.25 [-0.70, 0.21]
PH: Healthier	-0.32 [-0.80, 0.16]	-0.40 [-0.87, 0.07]	-0.26 [-0.71, 0.20]	-0.22 [-0.69, 0.26]	-0.27 [-0.73, 0.19]	-0.32 [-0.80, 0.16]
PH: A lot healthier	-0.31 [-0.84, 0.22]	-0.37 [-0.89, 0.15]	-0.16 [-0.67, 0.35]	-0.17 [-0.69, 0.36]	-0.20 [-0.72, 0.31]	-0.28 [-0.82, 0.25]
PH: Never ill	-0.04 [-0.94, 0.87]	-0.15 [-1.04, 0.74]	0.11 [-0.76, 0.98]	0.10 [-0.79, 0.99]	0.09 [-0.79, 0.96]	-0.17 [-1.07, 0.74]
Diagnosed mental disorder: yes	0.19 [-0.09, 0.47]	0.20 [-0.07, 0.47]	0.09 [-0.18, 0.36]	0.11 [-0.16, 0.39]	0.19 [-0.08, 0.46]	0.29 [0.01, 0.56]
COVID-19 riskgroup: no	0.01 [-0.31, 0.32]	0.11 [-0.20, 0.43]	0.12 [-0.18, 0.42]	0.04 [-0.26, 0.35]	0.03 [-0.27, 0.34]	0.03 [-0.29, 0.35]
COVID-19 riskgroup: Unsure	0.11 [-0.40, 0.61]	0.23 [-0.26, 0.72]	0.26 [-0.22, 0.74]	0.20 [-0.29, 0.69]	0.18 [-0.30, 0.66]	0.17 [-0.34, 0.67]
Num.Obs.	569	569	569	569	569	569

R2	0.123	0.156	0.193	0.160	0.183	0.113
R2 Adj.	0.096	0.130	0.168	0.134	0.158	0.085

Note. Multiple regression analyses were run individually for all RFs. 99 % confidence intervals in parentheses. Abbreviations: PAS_p, process-focused positive appraisal style; PSS, perceived social support; CSS, Corona-related social support; OPT, optimism; GSE, general self-efficacy; REC, perceived good stress recovery; NEU, neuroticism; PAC, positive appraisal of the Corona crisis; PH, perceived health condition.

*p<0.0028.

Table S6

Associations between hypothesized general resilience and risk factors (RFs) and general stressor reactivity (SR_G), second part

SKI	**-0.29<br>[-0.39, -0.18] ***							
COH		**-0.16<br>[-0.27, -0.06] ***						
MIN			**-0.30<br>[-0.40, -0.19] ***					
SJU				**0.28<br>[0.17, 0.38] ***				
ISO					**0.30<br>[0.19, 0.40] ***			
OVI						**0.33<br>[0.22, 0.43] ***		
SCO							**-0.40<br>[-0.50, -0.29] ***	
SCR								**0.35<br>[0.24, 0.45] ***
Age	-0.00 [-0.01, 0.01]	-0.00 [-0.01, 0.01]	-0.00 [-0.01, 0.01]	0.00 [-0.01, 0.01]	0.00 [-0.01, 0.01]	0.00 [-0.01, 0.01]	0.00 [-0.01, 0.01]	0.00 [-0.01, 0.01]
Gender: Female	0.21 [-0.09, 0.50]	0.27 [-0.04, 0.57]	0.17 [-0.13, 0.46]	0.20 [-0.10, 0.49]	0.23 [-0.06, 0.53]	0.17 [-0.12, 0.47]	0.18 [-0.11, 0.46]	0.24 [-0.05, 0.53]
Relationship status: Other	0.24 [-0.07, 0.55]	0.24 [-0.08, 0.56]	0.23 [-0.08, 0.54]	0.28 [-0.03, 0.59]	0.26 [-0.05, 0.57]	0.26 [-0.05, 0.56]	0.24 [-0.06, 0.54]	0.25 [-0.06, 0.56]
Relationship status: Single	0.15 [-0.13, 0.43]	0.10 [-0.20, 0.39]	0.12 [-0.16, 0.41]	0.11 [-0.17, 0.39]	0.01 [-0.27, 0.30]	0.08 [-0.20, 0.36]	0.10 [-0.17, 0.37]	0.07 [-0.21, 0.35]
People in household: 2	-0.26 [-0.63, 0.12]	-0.27 [-0.66, 0.12]	-0.30 [-0.67, 0.08]	-0.30 [-0.68, 0.08]	-0.26 [-0.64, 0.11]	-0.30 [-0.68, 0.07]	-0.24 [-0.61, 0.12]	-0.23 [-0.60, 0.14]
People in household: 3-4	**-0.45 [-0.81, -0.08] ***	-0.43 [-0.81, -0.06]	**-0.44 [-0.80, -0.08] ***	**-0.50 [-0.87, -0.14] ***	**-0.44 [-0.80, -0.08] ***	**-0.45 [-0.81, -0.09] ***	**-0.41 [-0.76, -0.06] ***	**-0.43 [-0.79, -0.08] ***
People in household: 5-6	-0.50 [-0.97, -0.03]	-0.48 [-0.97, 0.00]	-0.40 [-0.88, 0.07]	-0.50 [-0.98, -0.03]	-0.47 [-0.94, 0.00]	-0.48 [-0.95, -0.02]	-0.42 [-0.87, 0.04]	-0.40 [-0.87, 0.06]
People in household: >6	-0.31 [-1.19, 0.56]	-0.39 [-1.29, 0.51]	-0.32 [-1.19, 0.56]	-0.49 [-1.36, 0.39]	-0.49 [-1.36, 0.38]	-0.39 [-1.26, 0.47]	-0.24 [-1.08, 0.61]	-0.46 [-1.32, 0.39]
Clinical experience (yrs)	0.04 [-0.08, 0.15]	0.01 [-0.10, 0.13]	0.04 [-0.08, 0.16]	0.02 [-0.10, 0.13]	0.01 [-0.11, 0.12]	0.02 [-0.10, 0.13]	0.04 [-0.07, 0.15]	0.05 [-0.07, 0.16]
PH: Equally healthy	-0.22 [-0.67, 0.22]	-0.19 [-0.65, 0.27]	-0.20 [-0.64, 0.24]	-0.22 [-0.67, 0.22]	-0.15 [-0.59, 0.30]	-0.24 [-0.67, 0.20]	-0.12 [-0.54, 0.31]	-0.16 [-0.60, 0.27]
PH: Healthier	-0.32 [-0.79, 0.14]	-0.29 [-0.77, 0.20]	-0.30 [-0.77, 0.16]	-0.30 [-0.77, 0.17]	-0.20 [-0.67, 0.27]	-0.30 [-0.76, 0.16]	-0.19 [-0.64, 0.26]	-0.24 [-0.71, 0.22]
PH: A lot healthier	-0.23 [-0.75, 0.29]	-0.29 [-0.83, 0.25]	-0.21 [-0.73, 0.31]	-0.24 [-0.76, 0.29]	-0.15 [-0.67, 0.37]	-0.21 [-0.72, 0.31]	-0.09 [-0.59, 0.42]	-0.16 [-0.67, 0.36]
PH: Never ill	-0.08 [-0.97, 0.80]	-0.04 [-0.96, 0.87]	0.03 [-0.86, 0.91]	-0.11 [-1.00, 0.78]	0.13 [-0.76, 1.02]	0.11 [-0.77, 0.99]	0.18 [-0.68, 1.04]	0.02 [-0.85, 0.89]
Diagnosed mental disorder: yes	0.19 [-0.08, 0.46]	0.27 [-0.01, 0.54]	0.20 [-0.07, 0.47]	0.17 [-0.11, 0.44]	0.22 [-0.05, 0.49]	0.14 [-0.13, 0.41]	0.10 [-0.16, 0.37]	0.16 [-0.11, 0.43]
COVID-19 riskgroup: no	0.08 [-0.23, 0.39]	0.06 [-0.26, 0.37]	0.02 [-0.29, 0.33]	0.11 [-0.20, 0.42]	0.08 [-0.23, 0.39]	0.06 [-0.25, 0.36]	0.10 [-0.20, 0.39]	0.09 [-0.21, 0.40]
COVID-19 riskgroup: Unsure	0.20 [-0.29, 0.69]	0.21 [-0.29, 0.71]	0.13 [-0.36, 0.62]	0.27 [-0.22, 0.77]	0.20 [-0.29, 0.68]	0.19 [-0.29, 0.67]	0.22 [-0.25, 0.69]	0.21 [-0.27, 0.69]

Num.Obs.	569	569	569	569	569	569	569	569
R2	0.160	0.109	0.161	0.154	0.163	0.176	0.221	0.188
R2 Adj.	0.134	0.082	0.135	0.128	0.137	0.151	0.197	0.162

Note. Multiple regression analyses were run individually for all RFs. 99 % confidence intervals in parentheses. Abbreviations: SKI, self-kindness; COH, common humanity, MIN, mindfulness; SJU, self-judgment; ISO, isolation; OVI, overidentification; SCO, self-compassion; SCR, self-criticism; PH, perceived health condition.

[a]The multiple regression analyses includes not only the self-compassion subscales SKI, COH, MIN, SJU, ISO, OVI but also the sum score (SCO). SCO will not be included in the elastic net analyses for reasons of multicollinearity.

*p<0.0028.

Table S7

Associations between hypothesized profession-relevant resilience and risk factors (RFs) and general stressor reactivity (SR_G)

TSE	**-0.20 [-0.31, -0.09] ***			
CER		**-0.18 [-0.29, -0.07] ***		
UNC			**0.19 [0.08, 0.30] ***	
COS				**-0.38 [-0.49, -0.28] ***
Age	-0.00 [-0.01, 0.01]	-0.00 [-0.01, 0.01]	-0.00 [-0.01, 0.01]	-0.00 [-0.01, 0.01]
Gender: Female	0.27 [-0.03, 0.57]	0.27 [-0.04, 0.57]	0.29 [-0.01, 0.59]	0.26 [-0.02, 0.55]
Relationship status: Other	0.22 [-0.10, 0.54]	0.24 [-0.08, 0.56]	0.29 [-0.03, 0.61]	0.25 [-0.05, 0.55]
Relationship status: Single	0.07 [-0.22, 0.36]	0.11 [-0.19, 0.40]	0.09 [-0.20, 0.38]	0.17 [-0.11, 0.44]
People in household: 2	-0.33 [-0.72, 0.06]	-0.27 [-0.66, 0.12]	-0.29 [-0.68, 0.10]	-0.16 [-0.52, 0.21]
People in household: 3-4	**-0.49 [-0.86, -0.12] ***	**-0.45 [-0.82, -0.07] ***	**-0.46 [-0.83, -0.09] ***	-0.29 [-0.64, 0.07]
People in household: 5-6	-0.54 [-1.03, -0.06]	-0.49 [-0.97, -0.01]	-0.48 [-0.96, 0.00]	-0.30 [-0.76, 0.16]
People in household: >6	-0.45 [-1.35, 0.45]	-0.47 [-1.37, 0.43]	-0.47 [-1.37, 0.42]	-0.14 [-0.99, 0.72]
Clinical experience (yrs)	0.04 [-0.08, 0.16]	0.02 [-0.10, 0.14]	0.02 [-0.10, 0.14]	0.06 [-0.06, 0.17]
PH: Equally healthy	-0.29 [-0.74, 0.16]	-0.22 [-0.67, 0.23]	-0.23 [-0.68, 0.22]	-0.25 [-0.68, 0.17]
PH: Healthier	-0.37 [-0.84, 0.11]	-0.30 [-0.77, 0.18]	-0.31 [-0.79, 0.16]	-0.30 [-0.75, 0.15]
PH: A lot healthier	-0.31 [-0.83, 0.22]	-0.26 [-0.80, 0.27]	-0.28 [-0.81, 0.25]	-0.16 [-0.67, 0.34]
PH: Never ill	-0.10 [-1.01, 0.80]	-0.03 [-0.94, 0.88]	-0.02 [-0.93, 0.89]	-0.05 [-0.90, 0.81]
Diagnosed mental disorder: yes	0.28 [-0.00, 0.55]	0.29 [0.01, 0.56]	0.27 [-0.01, 0.55]	0.23 [-0.03, 0.49]
COVID-19 riskgroup: no	0.08 [-0.24, 0.39]	0.07 [-0.25, 0.39]	0.06 [-0.25, 0.38]	0.06 [-0.24, 0.36]
COVID-19 riskgroup: Unsure	0.19 [-0.31, 0.69]	0.23 [-0.27, 0.73]	0.23 [-0.27, 0.73]	0.19 [-0.28, 0.66]
Num.Obs.	569	569	569	569
R2	0.120	0.115	0.118	0.214
R2 Adj.	0.093	0.088	0.091	0.189

Note. Multiple regression analyses were run individually for all RFs. 99 % confidence intervals in parentheses. Abbreviations: TSE, self-efficacy as a therapist; CER, certainty about mental states; UNC, uncertainty about mental states; COS, compassion satisfaction; PH, perceived health condition. *p<0.0028.

Table S8

Associations between hypothesized general resilience and risk factors (RFs) and profession-specific stressor reactivity (SR_S), first part

PAS_p	-0.09 [-0.22, 0.04]					
PSS		**-0.33 [-0.44, -0.22] ***				
OPT			**-0.33 [-0.44, -0.23] ***			
REC				**-0.31 [-0.42, -0.19] ***		
NEU					**0.32 [0.21, 0.43] ***	
PAC						-0.10 [-0.21, 0.01]
Age	-0.00 [-0.01, 0.01]	-0.00 [-0.01, 0.01]	-0.00 [-0.01, 0.01]	0.00 [-0.01, 0.01]	0.00 [-0.01, 0.01]	-0.00 [-0.01, 0.01]
Gender: Female	0.23 [-0.08, 0.54]	0.30 [0.01, 0.59]	0.31 [0.02, 0.60]	0.15 [-0.15, 0.44]	0.07 [-0.23, 0.37]	0.26 [-0.04, 0.57]
Relationship status: Other	0.16 [-0.17, 0.48]	0.10 [-0.21, 0.41]	0.14 [-0.17, 0.44]	0.18 [-0.13, 0.49]	0.22 [-0.09, 0.53]	0.17 [-0.15, 0.50]
Relationship status: Single	0.13 [-0.17, 0.42]	0.07 [-0.21, 0.35]	0.06 [-0.22, 0.34]	0.05 [-0.23, 0.33]	0.17 [-0.12, 0.45]	0.11 [-0.18, 0.41]
People in household: 2	-0.17 [-0.57, 0.22]	-0.03 [-0.41, 0.35]	-0.18 [-0.56, 0.19]	-0.17 [-0.55, 0.21]	-0.18 [-0.55, 0.20]	-0.18 [-0.58, 0.21]
People in household: 3-4	-0.30 [-0.68, 0.08]	-0.15 [-0.52, 0.21]	-0.29 [-0.65, 0.07]	-0.27 [-0.64, 0.09]	-0.22 [-0.58, 0.15]	-0.31 [-0.69, 0.07]
People in household: 5-6	-0.56 [-1.05, -0.07]	-0.43 [-0.89, 0.04]	-0.45 [-0.92, 0.01]	-0.50 [-0.97, -0.03]	-0.50 [-0.97, -0.03]	-0.56 [-1.05, -0.07]
People in household: >6	-0.09 [-1.00, 0.83]	-0.22 [-1.09, 0.64]	-0.07 [-0.94, 0.79]	-0.14 [-1.02, 0.73]	-0.04 [-0.91, 0.83]	-0.09 [-1.00, 0.82]
Clinical experience (yrs)	0.02 [-0.11, 0.14]	0.00 [-0.12, 0.12]	0.02 [-0.09, 0.14]	0.05 [-0.07, 0.17]	0.03 [-0.09, 0.14]	0.02 [-0.10, 0.14]
PH: Equally healthy	-0.25 [-0.71, 0.21]	-0.31 [-0.74, 0.13]	-0.24 [-0.68, 0.20]	-0.18 [-0.63, 0.26]	-0.19 [-0.63, 0.25]	-0.25 [-0.71, 0.21]
PH: Healthier	-0.33 [-0.81, 0.16]	-0.38 [-0.84, 0.08]	-0.24 [-0.71, 0.23]	-0.19 [-0.66, 0.28]	-0.26 [-0.72, 0.21]	-0.32 [-0.81, 0.16]
PH: A lot healthier	-0.33 [-0.87, 0.21]	-0.37 [-0.88, 0.14]	-0.17 [-0.69, 0.35]	-0.16 [-0.69, 0.36]	-0.21 [-0.72, 0.31]	-0.31 [-0.85, 0.23]
PH: Never ill	-0.15 [-1.07, 0.78]	-0.19 [-1.06, 0.69]	0.05 [-0.83, 0.93]	0.07 [-0.82, 0.96]	0.03 [-0.85, 0.92]	-0.21 [-1.13, 0.72]
Diagnosed mental disorder: yes	0.27 [-0.02, 0.55]	0.20 [-0.07, 0.47]	0.11 [-0.16, 0.39]	0.13 [-0.15, 0.40]	0.21 [-0.06, 0.48]	0.31 [0.03, 0.59]
COVID-19 riskgroup: no	-0.22 [-0.55, 0.10]	-0.13 [-0.43, 0.18]	-0.14 [-0.44, 0.17]	-0.21 [-0.52, 0.10]	-0.22 [-0.53, 0.09]	-0.22 [-0.54, 0.11]

COVID-19 riskgroup: Unsure	-0.10 [-0.61, 0.41]	-0.02 [-0.50, 0.47]	-0.00 [-0.49, 0.48]	-0.06 [-0.55, 0.43]	-0.07 [-0.56, 0.41]	-0.08 [-0.59, 0.43]
Num.Obs.	569	569	569	569	569	569
R2	0.086	0.178	0.174	0.155	0.167	0.088
R2 Adj.	0.057	0.152	0.149	0.129	0.141	0.060

Note. Multiple regression analyses were run individually for all RFs. 99 % confidence intervals in parentheses. Abbreviations: PAS_p, process-focused positive appraisal style; PSS, perceived social support; CSS, Corona-related social support; OPT, optimism; GSE, general self-efficacy; REC, perceived good stress recovery; NEU, neuroticism; PAC, positive appraisal of the Corona crisis; PH, perceived health condition.

*p<0.0028.

Table S9

Associations between hypothesized general resilience and risk factors (RFs) and profession-specific stressor reactivity (SR_S), second part

SKI	**-0.19<br>[-0.30, -0.08] ***							
COH		-0.08<br>[-0.19, 0.02]						
MIN			**-0.21<br>[-0.32, -0.10] ***					
SJU				**0.35<br>[0.25, 0.46] ***				
ISO					**0.31<br>[0.21, 0.42] ***			
OVI						**0.37<br>[0.27, 0.48] ***		
SCO							**-0.37<br>[-0.48, -0.27] ***	
SCR								**0.37<br>[0.26, 0.47] ***
Age	-0.00 [-0.01, 0.01]	-0.00 [-0.01, 0.01]	0.00 [-0.01, 0.01]	0.00 [-0.01, 0.01]	0.00 [-0.01, 0.01]	0.00 [-0.01, 0.02]	0.00 [-0.01, 0.01]	0.00 [-0.01, 0.02]
Gender: Female	0.21 [-0.09, 0.52]	0.25 [-0.06, 0.56]	0.18 [-0.12, 0.49]	0.18 [-0.11, 0.47]	0.22 [-0.07, 0.52]	0.15 [-0.14, 0.44]	0.17 [-0.12, 0.46]	0.23 [-0.05, 0.52]
Relationship status: Other	0.15 [-0.17, 0.47]	0.16 [-0.17, 0.48]	0.14 [-0.18, 0.46]	0.19 [-0.11, 0.50]	0.17 [-0.14, 0.48]	0.16 [-0.14, 0.46]	0.15 [-0.16, 0.45]	0.15 [-0.15, 0.46]
Relationship status: Single	0.16 [-0.13, 0.46]	0.13 [-0.17, 0.42]	0.15 [-0.14, 0.44]	0.15 [-0.13, 0.43]	0.04 [-0.24, 0.33]	0.11 [-0.16, 0.39]	0.13 [-0.14, 0.41]	0.10 [-0.17, 0.38]
People in household: 2	-0.15 [-0.54, 0.24]	-0.16 [-0.56, 0.23]	-0.18 [-0.56, 0.21]	-0.17 [-0.54, 0.21]	-0.14 [-0.51, 0.24]	-0.17 [-0.54, 0.20]	-0.12 [-0.49, 0.25]	-0.10 [-0.47, 0.27]
People in household: 3-4	-0.28 [-0.66, 0.09]	-0.28 [-0.66, 0.10]	-0.28 [-0.65, 0.09]	-0.33 [-0.69, 0.03]	-0.26 [-0.62, 0.10]	-0.26 [-0.62, 0.09]	-0.24 [-0.59, 0.12]	-0.26 [-0.61, 0.10]
People in household: 5-6	**-0.57 [-1.05, -0.08] ***	-0.56 [-1.05, -0.07]	-0.50 [-0.98, -0.01]	**-0.56 [-1.02, -0.10] ***	-0.52 [-0.99, -0.05]	**-0.53 [-0.99, -0.07] ***	-0.48 [-0.94, -0.02]	-0.46 [-0.92, 0.01]
People in household: >6	0.01 [-0.89, 0.91]	-0.06 [-0.98, 0.86]	0.02 [-0.87, 0.92]	-0.07 [-0.92, 0.79]	-0.08 [-0.95, 0.79]	0.04 [-0.82, 0.89]	0.15 [-0.70, 1.01]	-0.05 [-0.91, 0.80]
Clinical experience (yrs)	0.03 [-0.09, 0.16]	0.02 [-0.11, 0.14]	0.04 [-0.08, 0.16]	0.03 [-0.09, 0.14]	0.02 [-0.10, 0.13]	0.03 [-0.09, 0.14]	0.04 [-0.07, 0.16]	0.06 [-0.06, 0.17]
PH: Equally healthy	-0.24 [-0.69, 0.22]	-0.23 [-0.69, 0.23]	-0.22 [-0.67, 0.23]	-0.20 [-0.64, 0.23]	-0.13 [-0.58, 0.31]	-0.22 [-0.66, 0.21]	-0.12 [-0.55, 0.31]	-0.15 [-0.58, 0.28]
PH: Healthier	-0.32 [-0.80, 0.16]	-0.31 [-0.79, 0.18]	-0.30 [-0.78, 0.18]	-0.26 [-0.72, 0.19]	-0.17 [-0.63, 0.30]	-0.27 [-0.73, 0.19]	-0.18 [-0.63, 0.28]	-0.21 [-0.67, 0.24]

PH: A lot healthier	-0.27 [-0.80, 0.27]	-0.32 [-0.86, 0.22]	-0.25 [-0.78, 0.28]	-0.20 [-0.71, 0.30]	-0.13 [-0.65, 0.39]	-0.18 [-0.69, 0.33]	-0.10 [-0.61, 0.41]	-0.14 [-0.65, 0.37]
PH: Never ill	-0.15 [-1.06, 0.76]	-0.14 [-1.07, 0.78]	-0.07 [-0.98, 0.84]	-0.13 [-1.00, 0.73]	0.11 [-0.78, 1.00]	0.11 [-0.75, 0.98]	0.12 [-0.75, 0.99]	-0.01 [-0.88, 0.86]
Diagnosed mental disorder: yes	0.24 [-0.04, 0.52]	0.30 [0.02, 0.58]	0.24 [-0.04, 0.52]	0.15 [-0.12, 0.41]	0.23 [-0.04, 0.50]	0.13 [-0.13, 0.40]	0.13 [-0.14, 0.40]	0.17 [-0.10, 0.43]
COVID-19 riskgroup: no	-0.18 [-0.50, 0.13]	-0.20 [-0.52, 0.12]	-0.23 [-0.54, 0.09]	-0.12 [-0.43, 0.18]	-0.17 [-0.48, 0.14]	-0.19 [-0.50, 0.11]	-0.16 [-0.46, 0.14]	-0.16 [-0.46, 0.15]
COVID-19 riskgroup: Unsure	-0.06 [-0.56, 0.45]	-0.05 [-0.56, 0.46]	-0.11 [-0.61, 0.39]	0.04 [-0.44, 0.52]	-0.06 [-0.54, 0.43]	-0.06 [-0.54, 0.41]	-0.04 [-0.52, 0.44]	-0.04 [-0.52, 0.44]
Num.Obs.	569	569	569	569	569	569	569	569
R2	0.111	0.085	0.117	0.190	0.167	0.199	0.198	0.192
R2 Adj.	0.084	0.056	0.090	0.165	0.142	0.174	0.174	0.167

Note. Multiple regression analyses were run individually for all RFs. 99 % confidence intervals in parentheses. Abbreviations: SKI, self-kindness; COH, common humanity, MIN, mindfulness; SJU, self-judgment; ISO, isolation; OVI, overidentification; SCO, self-compassion; SCR, self-criticism; PH, perceived health condition.

[a]The multiple regression analyses includes not only the self-compassion subscales SKI, COH, MIN, SJU, ISO, OVI but also the sumscore (SCO). SCO will not be included in the elastic net analyses for reasons of multicollinearity.

*p<0.0028.

Table S10

Associations between hypothesized profession-relevant resilience and risk factors (RFs) and profession-specific stressor reactivity (SR_S)

TSE	**-0.29 [-0.41, -0.18] ***			
CER		**-0.27 [-0.38, -0.16] ***		
UNC			**0.25 [0.14, 0.36] ***	
COS				**-0.39 [-0.50, -0.28] ***
Age	0.00 [-0.01, 0.01]	0.00 [-0.01, 0.01]	-0.00 [-0.01, 0.01]	0.00 [-0.01, 0.01]
Gender: Female	0.27 [-0.02, 0.57]	0.26 [-0.03, 0.56]	0.29 [-0.01, 0.59]	0.25 [-0.03, 0.53]
Relationship status: Other	0.09 [-0.22, 0.41]	0.14 [-0.18, 0.45]	0.20 [-0.12, 0.51]	0.14 [-0.16, 0.44]
Relationship status: Single	0.10 [-0.19, 0.38]	0.15 [-0.14, 0.43]	0.13 [-0.16, 0.41]	0.20 [-0.07, 0.48]
People in household: 2	-0.21 [-0.59, 0.17]	-0.12 [-0.50, 0.26]	-0.15 [-0.54, 0.23]	-0.03 [-0.40, 0.34]
People in household: 3-4	-0.31 [-0.68, 0.05]	-0.25 [-0.62, 0.12]	-0.27 [-0.64, 0.09]	-0.11 [-0.46, 0.25]
People in household: 5-6	**-0.61 [-1.08, -0.14] ***	-0.52 [-1.00, -0.05]	-0.52 [-1.00, -0.04]	-0.35 [-0.81, 0.11]
People in household: >6	0.01 [-0.86, 0.89]	-0.02 [-0.90, 0.86]	-0.03 [-0.92, 0.85]	0.28 [-0.57, 1.13]
Clinical experience (yrs)	0.06 [-0.05, 0.18]	0.03 [-0.09, 0.15]	0.03 [-0.09, 0.15]	0.07 [-0.05, 0.18]
PH: Equally healthy	-0.30 [-0.74, 0.14]	-0.20 [-0.64, 0.25]	-0.22 [-0.66, 0.23]	-0.26 [-0.69, 0.16]
PH: Healthier	-0.35 [-0.82, 0.11]	-0.25 [-0.72, 0.22]	-0.29 [-0.76, 0.19]	-0.29 [-0.74, 0.16]
PH: A lot healthier	-0.29 [-0.81, 0.23]	-0.22 [-0.75, 0.30]	-0.26 [-0.78, 0.27]	-0.17 [-0.67, 0.33]
PH: Never ill	-0.13 [-1.01, 0.76]	-0.01 [-0.91, 0.88]	-0.03 [-0.93, 0.87]	-0.10 [-0.96, 0.75]
Diagnosed mental disorder: yes	0.28 [0.01, 0.55]	0.30 [0.03, 0.57]	0.27 [-0.00, 0.54]	0.23 [-0.03, 0.49]
COVID-19 riskgroup: no	-0.16 [-0.47, 0.15]	-0.17 [-0.49, 0.14]	-0.18 [-0.49, 0.14]	-0.19 [-0.49, 0.11]
COVID-19 riskgroup: Unsure	-0.06 [-0.55, 0.43]	-0.01 [-0.51, 0.48]	-0.02 [-0.51, 0.48]	-0.06 [-0.53, 0.41]
Num.Obs.	569	569	569	569
R2	0.155	0.146	0.139	0.213
R2 Adj.	0.129	0.119	0.113	0.189

Note. Multiple regression analyses were run individually for all RFs. 99 % confidence intervals in parentheses. Abbreviations: TSE, self-efficacy as a therapist; CER, certainty about mental states; UNC, uncertainty about mental states; COS, compassion satisfaction; PH, perceived health condition.

*$p<0.0028$.

Table S11

Regularized beta coefficients of resilience and risk factors (RFs) and covariates

**Variable**	**Regularized beta coefficient**		
	**SR_G; α= 0.03**	**SR_S; α= 0.03**	**SR_S; α= 0.36**
PAS_p	-0.01	0.09	0.1
PSS	-0.08	-0.15	-0.16
OPT	-0.10	-0.11	-0.10
REC	-0.07	-0.1	-0.1
NEU	0.11	0.09	0.09
PAC	-0.05	0.01	0.01
SCO	-0.12	0.07	-0.06
SCR	0.05	0.06	0.05
TSE	0.01	-0.08	-0.08
CER	0.04	-0.03	-0.02
UNC	0.05	0.08	0.09
COS	-0.20	-0.15	-0.16
Age	0.01	-0.03	0.03
Gender: Female	0.13	0.12	0.16
Relationship status: Other	0.17	0.06	0.05
Relationshipstatus: Single	0.10	0.06	0.05
People in household: 2	-0.02	0.03	-
People in household: 3-4	-0.13	-0.02	-0.02
People in household: 5-6	-0.04	-0.11	-0.15
Clinical experience (yrs)	0.06	0.07	0.06
PH: Equally healthy	-0.08	-0.07	-0.08
PH: Healthier	-0.09	-0.06	-0.07
PH: A lot healthier	0.05	0.03	-
PH: Never ill	0.07	0.02	-
Diagnosed mental disorder: yes	-	0.02	-
COVID-19 risk group: no	0.04	-0.06	-0.05
COVID-19 risk group: Unsure	0.08	0.01	-

Note. Combined multi-variable analysis (elastic net) of relative associations between RFs and stressor reactivity (SR) was conducted separately for both general (SR_G) and profession-specific

(SR_S) stressor reactivity at an alpha value of α=0.03, reflecting greater similarity to ridge than LASSO regression. Optimal lambda values (λ + 1 SE) were λ=0.05 for general and λ=0.1 for profession-specific stressor reactivity. For SR_S coefficients are also shown for the alpha value of α=0.36, determined by cross-validation, at an optimal lambda value of λ=0.02. Coefficients of profession-relevant factors are placed on the right. Abbreviations: PAS_P, process-focused positive appraisal style; PSS, perceived social support; OPT, optimism; GSE, general self-efficacy; REC, perceived good stress recovery; NEU, neuroticism; PAC, positive appraisal of the Corona crisis; SCO, self-compassion; SCR, self-criticism; TSE, self-efficacy as a therapist; CER, certainty about mental states; UNC, uncertainty about mental states; COS, compassion satisfaction, PH, perceived health condition.

References

Beierlein, C., Kovaleva, A., Kemper, C. J., Rammstedt, B. (2012). *ASKU - Allgemeine Selbstwirksamkeit Kurzskala. Messinstrument zur Erfassung subjektiver Kompetenzerwartungen.* https://doi.org/10.23668/psycharchives.418

Blatt, S. J., D'Afflitti, J. P., & Quinlan, D. M. (1976). *Depressive Experiences Questionnaire (DEQ)* [Database record]. APA PsycTests. https://doi.org/10.1037/t02165-000

Brenk-Franz, K., Ehrenthal, J., Freund, T., Schneider, N., Strauß, B., Tiesler, F., Schauenburg, H,. & Gensichen, J. (2018). Evaluation of the short form of “Experience in Close Relationships” (Revised, German Version “ECR-RD12”) -A tool to measure adult attachment in primary care. *PloS ONE*, *13*(1), e0191254.

Bride, B. E. (2013). *The Secondary Traumatic Stress Scale, DSM5 Revision*. [Unpublished manuscript].

Bride, B. E., Robinson, M. M., Yegidis, B., & Figley, C. R. (2004). Development and validation of the secondary traumatic stress scale. *Research on social work practice*, *14*(1), 27-35. https://doi.org/ 10.1177/1049731503254106

Bögemann, S., Puhlmann, L., Wackerhagen, C., Zerban, M., Riepenhausen, A., Köber, G., Yuen, K., Pooseh, S., Marciniak, M. A., Reppmann, Z., Uściłko, A., Weermeijer, J., Lenferink, D. B., Mituniewicz, J., Robak, N., Donner, N., Mestdagh, M., Verdonck, S., van Dick, R., … Kalisch, R. (2022). *Psychological resilience factors and their association with weekly stressor reactivity during the COVID-19 outbreak in Europe*. PsyArXiv. https://doi.org/10.31234/osf.io/f7sy3

Carver C. S. (1997). You want to measure coping but your protocol's too long: consider the brief COPE. *International Journal of Behavioral Medicine*, *4*(1), 92–100. https://doi.org/10.1207/s15327558ijbm0401_6

Dunkel, D., Antretter, E., Fröhlich-Walser, S., & Haring, C. (2005). Evaluation der Kurzform des Fragebogens zur Sozialen Unterstützung (SOZU-K-22) in klinischen und nichtklinischen Stichproben [Evaluation of the short-form social support questionnaire (SOZU-K-22) in clinical and non-clinical samples]. *Psychotherapie, Psychosomatik, medizinische Psychologie*, *55*(5), 266–277. https://doi.org/10.1055/s-2004-834746

Fonagy, P., Luyten, P., Moulton-Perkins, A., Lee, Y. W., Warren, F., Howard, S., Ghinai R., Fearon P., & Lowyck, B. (2016). Development and validation of a self-report measure of mentalizing: The reflective functioning questionnaire. *PLoS ONE*, *11*(7), e0158678. https://doi.org/10.1371/journal.pone.0158678

Friedman, J., Hastie, T., & Tibshirani, R. (2010). Regularization Paths for Generalized Linear Models via Coordinate Descent. *Journal of Statistical Software*, *33*(1), 1–22.

Garnefski, N., & Kraaij, V. (2006). Cognitive emotion regulation questionnaire - development of a short 18-item version (CERQ-short). *Personality and Individual Differences, 41*(6), 1045–1053. https://doi.org/10.1016/j.paid.2006.04.010

Goldberg, D. P., Gater, R., Sartorius, N., Ustun, T. B., Piccinelli, M., Gureje, O.,& Rutter, C. (1997) The validity of two versions of the GHQ in the WHO study of mental illness in general health care. *Psychological Medicine*, *27*(1), 191–197. https://doi.org/10.1017/s0033291796004242

Hastie, T., Tibshirani, R., Wainwright, M. (2015). *Statistical learning with sparsity: the Lasso and generalizations*. Chapman & Hall/CRC Press, 2015. https://doi.org/10.1111/insr.12167

Jakobsen, J. C., Gluud, C., Wetterslev, J., & Winkel, P. (2017). When and how should multiple imputation be used for handling missing data in randomised clinical trials - a practical guide with flowcharts. *BMC Medical Research Methodology*, *17*(1), 162. https://doi.org/10.1186/s12874-017-0442-1

Krstajic, D., Buturovic, L. J., Leahy, D. E., & Thomas, S. (2014). Cross-validation pitfalls when selecting and assessing regression and classification models. *Journal of Cheminformatics*, *6*(1), 10. https://doi.org/10.1186/1758-2946-6-10

Little, R. J. A. (1988). A Test of Missing Completely at Random for Multivariate Data with Missing Values. *Journal of the American Statistical Association*, *83*(404), 1198–1202. https://doi.org/10.2307/2290157

Neff, K. D. (2003). The development and validation of a scale to measure self-compassion. *Self and Identity*, *2*(3), 223–250. https://doi.org/10.1080/15298860309027

Petri-Romão, P., Engen, H., Rupanova, A., Puhlmann, L., Zerban, M., Neumann, R. J., Malyshau, A., Ahrens, K. F., Schick, A., Kollmann, B., Walter, H., Plichta, M., Reif, A., Chmitorz, A., Tüscher, O., Basten U, & Kalisch, R. (2023). *Self-report assessment of Positive Appraisal Style (PAS): development of a process-focused and a content-focused questionnaire for use in mental health and resilience research.* PsyArXiv. https://doi.org/10.31234/osf.io/fpw94

Rammstedt B, John OP. Measuring personality in one minute or less: A 10-item short version of the Big Five Inventory in English and German. *Journal of Research in Personality* 2007; **41**: 203–212.

Rudich, Z., Lerman, S. F., Gurevich, B., Weksler, N., & Shahar, G. (2008). Patients' self-criticism is a stronger predictor of physician's evaluation of prognosis than pain diagnosis or severity in chronic pain patients. *The Journal of Pain*, *9*(3), 210–216. https://doi.org/10.1016/j.jpain.2007.10.013

Smith, B. W., Dalen, J., Wiggins, K., Tooley, E., Christopher, P., & Bernard, J. (2008). The brief resilience scale: assessing the ability to bounce back. *International Journal of Behavioral Medicine*, *15*(3), 194–200. https://doi.org/10.1080/10705500802222972

Stamm, B. H. (2010). *The concise ProQOL manual*. https://proqol.org/proqol-manual

van Buuren, S. (2018). *Flexible Imputation of Missing Data, Second Edition*. Chapman and Hall/CRC. https://doi.org/10.1201/9780429492259

Veer, I. M., Riepenhausen, A., Zerban, M., Wackerhagen, C., Puhlmann, L. M. C., Engen, H., Köber, G., Bögemann, S. A., Weermeijer, J., Uściłko, A., Mor, N., Marciniak, M. A., Askelund, A. D., Al-Kamel, A., Ayash, S., Barsuola, G., Bartkute-Norkuniene, V., Battaglia, S., Bobko, Y., … Kalisch, R. (2021). Psycho-social factors associated with mental resilience in the Corona lockdown. *Translational Psychiatry*, *11*(1), 67. https://doi.org/10.1038/s41398-020-01150-4

Wilkerson, A., & Basco, M. R. (2014). Therapists' self-efficacy for CBT dissemination: Is supervision the key?. *Journal of Psychology & Psychotherapy*, *4*(3), 1-6.
